# Trading Speed and Accuracy by Coding Time: A Coupled-circuit Cortical Model

**DOI:** 10.1371/journal.pcbi.1003021

**Published:** 2013-04-04

**Authors:** Dominic Standage, Hongzhi You, Da-Hui Wang, Michael C. Dorris

**Affiliations:** 1 Department of Biomedical and Molecular Sciences and Center for Neuroscience Studies, Queen's University, Kingston, Ontario, Canada; 2 Department of Systems Science and National Key Laboratory of Cognitive Neuroscience and Learning, Beijing Normal University, Beijing, China; 3 Institute of Neuroscience, Shanghai Institutes for Biological Sciences, Chinese Academy of Sciences, Shanghai, China; Indiana University, United States of America

## Abstract

Our actions take place in space and time, but despite the role of time in decision theory and the growing acknowledgement that the encoding of time is crucial to behaviour, few studies have considered the interactions between neural codes for objects in space and for elapsed time during perceptual decisions. The speed-accuracy trade-off (SAT) provides a window into spatiotemporal interactions. Our hypothesis is that temporal coding determines the rate at which spatial evidence is integrated, controlling the SAT by gain modulation. Here, we propose that local cortical circuits are inherently suited to the relevant spatial and temporal coding. In simulations of an interval estimation task, we use a generic local-circuit model to encode time by ‘climbing’ activity, seen in cortex during tasks with a timing requirement. The model is a network of simulated pyramidal cells and inhibitory interneurons, connected by conductance synapses. A simple learning rule enables the network to quickly produce new interval estimates, which show signature characteristics of estimates by experimental subjects. Analysis of network dynamics formally characterizes this generic, local-circuit timing mechanism. In simulations of a perceptual decision task, we couple two such networks. Network function is determined only by spatial selectivity and NMDA receptor conductance strength; all other parameters are identical. To trade speed and accuracy, the timing network simply learns longer or shorter intervals, driving the rate of downstream decision processing by spatially non-selective input, an established form of gain modulation. Like the timing network's interval estimates, decision times show signature characteristics of those by experimental subjects. Overall, we propose, demonstrate and analyse a generic mechanism for timing, a generic mechanism for modulation of decision processing by temporal codes, and we make predictions for experimental verification.

## Author Summary

Studies in neuroscience have characterized how the brain represents objects in space and how these objects are selected for detailed perceptual processing. The selection process entails a decision about which object is favoured by the available evidence over time. This period of time is typically in the range of hundreds of milliseconds and is widely believed to be crucial for decisions, allowing neurons to filter noise in the evidence. Despite the widespread belief that time plays this role in decisions and the growing recognition that the brain estimates elapsed time during perceptual tasks, few studies have considered how the encoding of time effects decision making. We propose that neurons encode time in this range by the same general mechanisms used to select objects for detailed processing, and that these temporal representations determine how long evidence is filtered. To this end, we simulate a perceptual decision by coupling two instances of a neural network widely used to simulate localized regions of the cerebral cortex. One network encodes the passage of time and the other makes decisions based on noisy evidence. The former influences the performance of the latter, reproducing signature characteristics of temporal estimates and perceptual decisions.

## Introduction

It is likely that the cerebral cortex evolved to provide a model of the world, serving decisions for action. Our actions take place in space and time and both of these dimensions are considered in the dominant hypothesis of decision making, where noisy spatial evidence is averaged over time (see [1]–[3]). The longer we spend averaging, the more accurate our decisions [4]. A trade-off between speed and accuracy is implicit in this framework and is a hallmark of decision tasks [5], but the mechanism by which we determine how long to spend averaging is an open question [6]. In recent years, there has been increasing acknowledgement that the encoding of time may be as crucial to behaviour as the encoding of space [7] and several studies have considered roles for temporal codes in decision making [8]–[11]. Under this approach, time is not a passive medium for spatial averaging, but is actively encoded during decisions, determining the rate at which they unfold. Accordingly, the speed-accuracy trade-off (SAT) can be controlled by the estimation of temporal intervals, determining how long spatial evidence is integrated [11].

Our ability to represent time covers at least twelve orders of magnitude, from the scale of microseconds to circadian rhythms, and different neural mechanisms are believed to support representations of vastly different temporal duration [12], [13]. Here, we focus on the hundreds of milliseconds range, the relevant order for the most well studied perceptual decision tasks [1], [3], [14]. Two fundamental questions in the study of temporal processing are whether the representation of time is centralized or distributed [15], [16], and whether the circuitry involved is specialized or generic [17], [18]. In this paper, we propose that local-circuit cortical processing is inherently suited to the representation of space and time on this order, supporting a distributed, generic processing framework. To this end, we demonstrate that a generic biophysical model of a local cortical circuit can estimate time in the hundreds of milliseconds range, where ‘climbing’ activity resembles that seen in cortex during tasks with a timing requirement and estimates of temporal intervals show signature characteristics of temporal estimates by experimental subjects. The network estimates different intervals by the scaling of a single term controlling local-circuit dynamics by the strength of NMDA receptor (NMDAR) conductance. Analysis of network dynamics formally characterizes this timing mechanism and a simple learning rule is sufficient for the network to quickly learn the intervals.

In simulations of a decision task, we couple two such generic networks with identical parameters except for the NMDAR scale factor. One network encodes elapsed time relative to a learned interval. The other decides which of two stimuli has more evidence. As climbing activity evolves in the timing network, it governs the rate of downstream decision processing by gain modulation. To trade speed and accuracy, the timing network simply imposes different temporal constraints on the decision network. The model's activity and behaviour are consistent with a large body of electrophysiological and behavioural data from timing and decision tasks, as well as the hypothesis that cortical circuitry is canonical (see [19]). In our opinion, these results should be expected of a generally uniform structure that evolved to provide a model for action in a spatiotemporal world.

## Methods

To address the hypotheses that generic local-circuit processing is sufficient to support timing in the hundreds of milliseconds range (see [17], [18]) and that these temporal codes control the speed and accuracy of decisions [11], we simulated each of two local cortical circuits with a spiking-neuron implementation [20]–[26] of a model widely used in population and firing rate simulations of cortical circuits [27]–[29]. This class of model assumes a columnar structure, where a spatial continuum of bell-shaped population codes (bumps) is supported by net excitation between adjacent columns and net inhibition between distal columns. To emphasize the robustness of the principles underlying our hypotheses, the only differences between the two networks were their stimulus-selectivity and the strength of NMDAR conductance. The scaling of NMDAR conductance is an established mechanism for controlling intrinsic dynamics in these and related models [21], [26], [30], [31] and a potential biological correlate is provided in the Discussion, but our hypothesis does not require this specific mechanism, *e.g.* scaling the strength of feedback inhibition is another approach. As described below, the timing network had stronger NMDAR conductance, but only the decision network was selective for stimuli. These differences were sufficient to determine each network's function as a timer or decision maker.

In simulations of an interval estimation task, the timing network received noisy current injection, simulating synaptic bombardment from upstream cortical regions, but did not receive any spatially-selective input. Strong NMDAR conductance at intrinsic synapses enabled a local sub-population of the network to undergo ‘climbing’ activity (activity buildup) and the time at which this activity reached a fixed threshold was the network's estimate of a given interval (see [32]). Only the timing network was used in this task.

**Figure 1:**
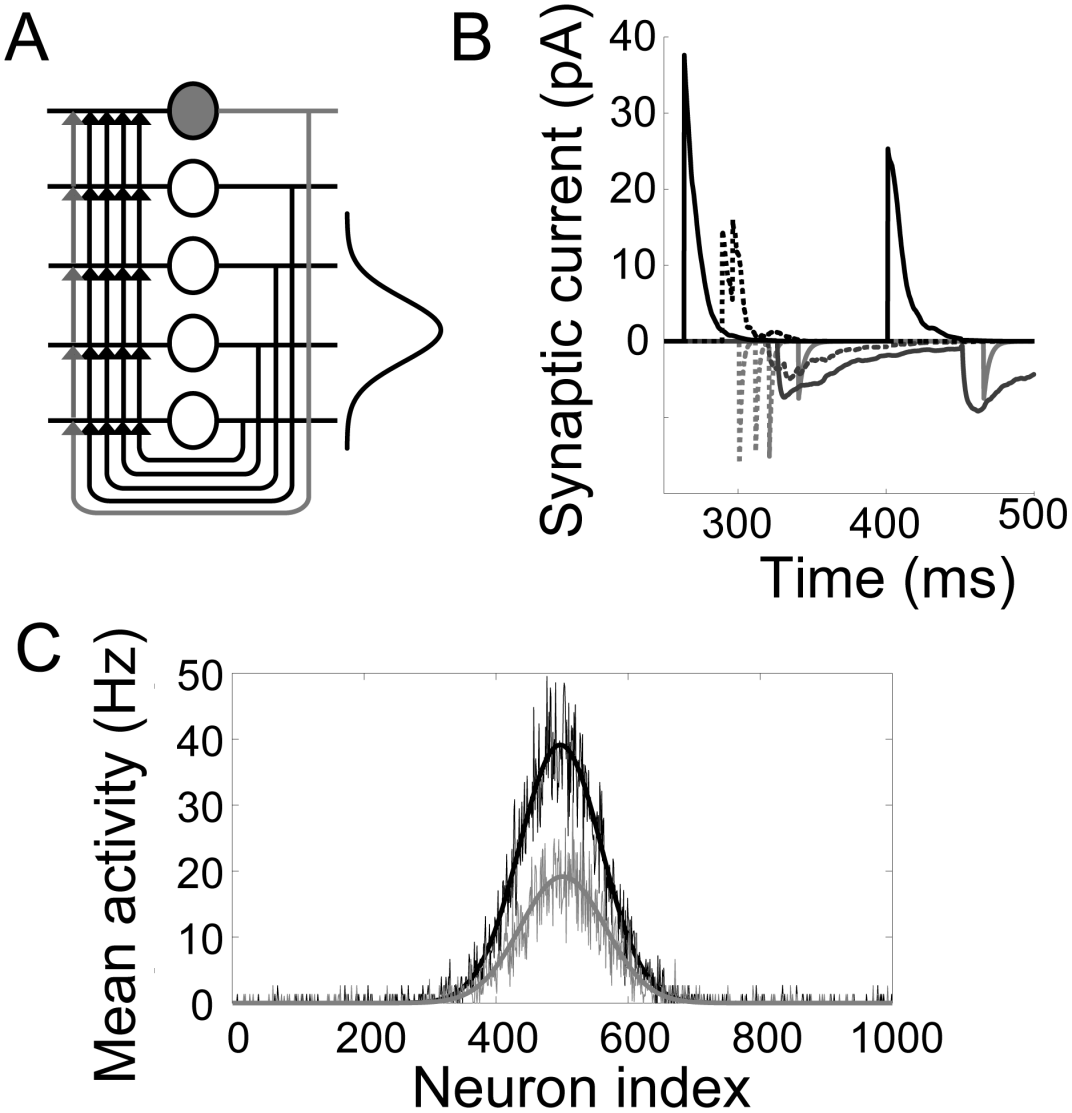
Local-circuit cortical model. (A) Fully recurrent network of pyramidal neurons (white circles with black projections) and inhibitory interneurons (grey filled circles with grey projections). The 4/1 ratio of pyramidal neurons to interneurons preserves the population sizes in the spiking model (1000/250). The strength of synaptic conductance between pyramidal cells is a Gaussian function of the spatial distance between them (Equation 1), depicted by the Gaussian curve on the right hand side. (B) Intrinsic AMPAR (negative, light grey), NMDAR (negative, dark grey) and GABAR (positive, black) currents onto a pyramidal neuron (solid curves) and onto an interneuron (dotted curves) during the background state. (C) Gain modulation of the decision network by the timing network (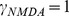) in a trial with only one stimulus for the purpose of demonstration. The stimulus was centred on pyramidal neuron 500. The grey curves show the mean spike density function (SDF, see Methods) over the downstream network during the first 250 ms of the trial. The black curves show the mean SDF over the last 250 ms. As climbing activity evolves in the timing network, the response to the stimulus in the downstream network increases without a change to stimulus selectivity. Smooth curves are Gaussian fits to the noisy curves.

In coupled-circuit simulations of a decision task, both networks received noisy current injection, but the decision network received two noisy, spatially-selective inputs and its task was to decide which was stronger. It also received spatially non-selective input from the timing network, *i.e.* every neuron in the timing network projected uniformly to every neuron in the decision network. Spatially non-selective input to recurrent networks is an established form of gain modulation [33], where the magnitude of a selective neural response is modulated by a second source of input (see [34], [35] and Figure 1C). Temporal constraints encoded by climbing activity upstream thereby modulated the rate of downstream decision processing. Note that we use the terms climbing activity and ascending activity interchangeably, and we use the term ramping to refer to ascending or descending activity (see the Discussion).

### The network model

The local circuit model is a fully recurrent network of leaky integrate-and-fire neurons [36], comprised of 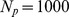 simulated pyramidal neurons and 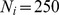 fast-spiking inhibitory interneurons. For pyramidal-to-pyramidal synapses, 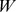 is a Gaussian function of the distance between neurons arranged in a ring. The weight 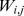 between any two pyramidal neurons 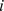 and 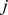 is therefore given by
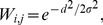
(1)where 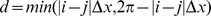 defines distance in the ring, 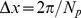 is a scale factor, and 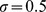, depicted on the right side of Figure 1A. The biological basis of 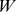 is the probability of lateral synaptic contact between pyramidal neurons, generally found to be monotonically decreasing over a distance of 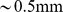 in layers 2/3 and 5 [37], [38]. Width parameter 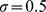 therefore corresponds to approximately 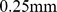 axially in cortical tissue, consistent with cortical tuning curves [39], [40]. Like earlier authors (e.g. [21], [24]), we do not attribute biological significance to the spatial periodicity of the network; rather, this arrangement allows the implementation of 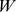 with all-to-all connectivity without biases due to asymmetric lateral interactions between pyramidal neurons. Synaptic connectivity from pyramidal neurons to interneurons, from interneurons to pyramidal neurons, and from interneurons to interneurons is unstructured in the network, so for each of these cases, 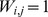 for all 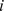 and 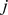.

Each model neuron is described by
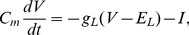
(2)where 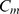 is the membrane capacitance of the neuron, 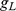 is the leakage conductance, 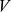 is the membrane potential, 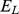 is the equilibrium potential, and 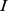 is the total input current. When 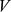 reaches a threshold 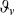, it is reset to 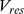, after which it is unresponsive to its input for an absolute refractory period of 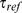. For pyramidal neurons, 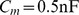, 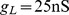, 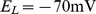, 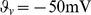, 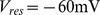 and 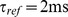. For interneurons, 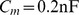, 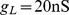, 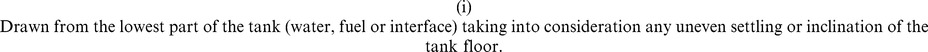, 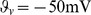, 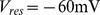 and 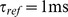 [21].

Excitatory currents from pyramidal neurons were mediated by AMPA receptor (AMPAR) and NMDAR conductances, and inhibitory currents from interneurons were mediated by GABA receptor (GABAR) conductances (Figure 1B). The total input current to each neuron 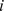 is given by
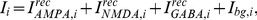
(3)where 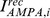, 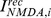 and 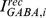 are the summed AMPAR, NMDAR and GABAR currents from intrinsic (recurrent) synapses, and 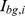 is background noise, described below. These intrinsic currents are defined by
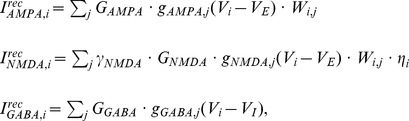
(4)where 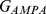, 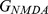 and 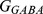 are the respective strengths of AMPAR, NMDAR and GABAR conductance, 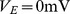 is the reversal potential for AMPARs and NMDARs and 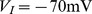 is the reversal potential for GABARs [21]. AMPAR and GABAR activation (proportion of open channels) are described by
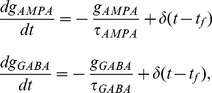
(5)where 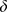 is the Dirac delta function and 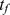 is the time of firing of a pre-synaptic neuron. For NMDAR activation, 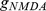 has a slower rise and decay and is described by
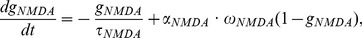
(6)where 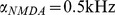 controls the saturation of NMDAR channels at high pre-synaptic spike frequencies [21]. The slower opening of NMDAR channels is captured by
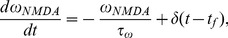
(7)where 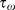 was set to 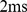 [21]. The voltage-dependence of NMDARs is captured by 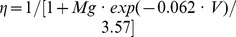, where 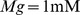 describes the extracellular Magnesium concentration and 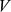 is measured in millivolts [41]. The scale factor 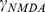 is described below.

The time constants and conductance strengths of AMPAR, NMDAR and GABAR synapses onto cortical pyramidal neurons and inhibitory interneurons vary according to tissue preparation, recording method, species, cortical layer, and to some degree, cortical area within a species or layer. *En masse*, electrophysiological data provide reasonable guidelines for these parameters, but we emphasize that nothing in the model is fine tuned and our results hold for a broad range of parameters. For AMPAR-mediated currents, 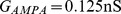 and 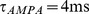 at synapses onto pyramidal neurons, and 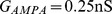 and 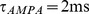 at synapses onto interneurons, producing fast-decaying monosynaptic AMPAR currents on the order of 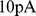 [42], [43] that are stronger and faster onto inhibitory interneurons than pyramidal neurons [44]–[46]. For NMDAR-mediated currents, 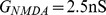 and 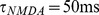 at synapses onto pyramidal neurons, and 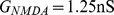 and 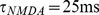 at synapses onto interneurons, producing slow-decaying monosynaptic NMDAR currents on the order of 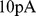 [42], [47] that are stronger and slower at synapses onto pyramidal neurons than interneurons [46]. Our excitatory synaptic parameters thus emphasize fast inhibitory recruitment in response to slower excitation (see [48] for discussion). For GABAR-mediated currents, 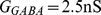 and 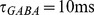 at synapses onto pyramidal neurons and 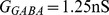 and 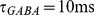 at synapses onto interneurons [49], [50], producing monosynaptic GABAR currents several times stronger than the above excitatory currents, where stronger conductance onto pyramidal cells was meant to reflect the greater prevalence of GABAR synapses onto pyramidal cells than interneurons [51]. See Figure 1B for exemplary synaptic currents in the model.

#### Background activity

For each neuron, current 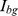 simulates *in vivo* cortical background activity by the point-conductance model of [52]:
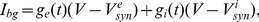
(8)where reversal potentials 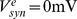 and 
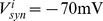
 are given the same values as those for excitatory and inhibitory synapses in Equation 4. The time-dependent excitatory and inhibitory conductances 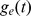 and 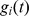 are updated at each timestep 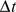 according to
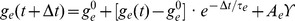
(9)and
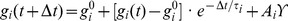
(10)where 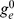 and 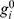 are average conductances, 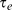 and 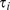 are time constants, 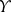 is normally distributed random noise with 0 mean and unit standard deviation. Amplitude coefficients 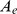 and 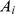 are defined by
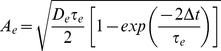
(11)and
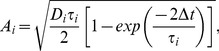
(12)where 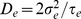 and 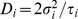 are noise ‘diffusion’ coefficients. See [52] for the derivation of these equations. We followed Table 1 of [52] for parameter values 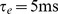, 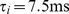, 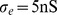 and 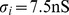 for pyramidal neurons and interneurons and 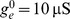 for pyramidal neurons. We gave nominal values to 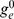 for interneurons and to 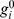 for pyramidal neurons and interneurons, setting these conductances to 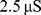, *i.e.* the network's intrinsic connectivity was sufficient to mediate realistic levels of inhibitory background activity onto pyramidal neurons and excitatory and inhibitory background activity onto interneurons.

**Table 1:** Parameters for the probability density of first passage times.

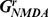	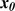	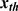	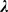
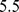	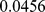	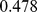	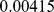
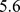	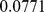	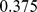	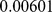
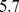	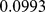	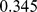	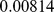
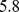	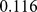	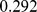	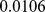
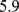	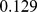	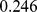	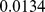
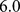	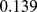	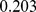	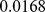
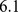	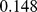	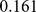	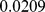
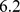	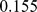	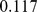	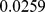

Parameter values for the probability density of first passage times, plotted in Figure 7. 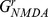: NMDAR conductance strength; 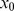: initial state; 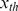: timing threshold in terms of NMDAR activation; 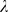: largest positive eigenvalue.

### Population activity

In both simulated tasks, performance was determined by the mean activity of localized populations of neurons. Spike density functions (SDF, rounded to the nearest millisecond) were therefore built for these neurons by convolving their spike trains with a rise-and-decay function
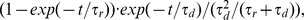
where t is the time following stimulus onset and 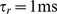 and 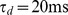 are the time constants of rise and decay respectively [53]. In the interval estimation task, it was necessary to first identify the relevant population (the bump population) before averaging its activity. To this end, we built SDFs for all pyramidal neurons in the network and the neuron with the highest mean SDF over the full trial was considered the centre of the bump. We included 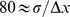 neurons on either side of this centre in the bump population. In the decision task, the centres of the response fields for the competing stimuli were pre-determined, so SDFs were constructed for these neurons, as well as the 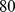 neurons on either side. All simulations were run with timestep 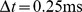 and the standard implementation of Euler's forward method.

### Reduction of the network to an integral and partial differential system

To investigate the mechanism by which the timing network produced climbing activity, we simplified the network to an equivalent integral and partial differential system using a Wilson-Cowan type approach [27], [54]. We then used methods for the study of non-linear dynamics and stochastic processes to analyse the reduced system.

Because pyramidal-to-pyramidal synaptic connectivity is structured and all synaptic connections with interneurons are unstructured, the firing rate of pyramidal neurons and interneurons can be modelled as
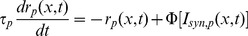
(13)and
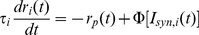
(14)where (as above) 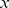 denotes spatial location and 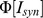 is the activation function
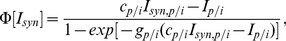
(15)with gain factor 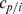 and noise factor 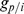. The synaptic current at pyramidal neurons at location 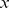 is 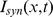 and consists of AMPAR-, NMDAR- and GABAR-mediated synaptic currents and background current, *i.e.* 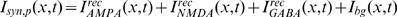. The first three of these currents can be approximated as
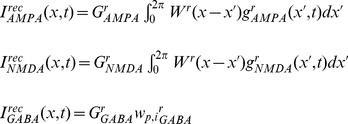
(16)where 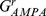, 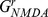 and 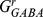 describe the effective synaptic strength. Superscripts ‘r’ denote the correspondence of terms in the ‘reduced’ system with those in the timing network. The synaptic currents onto interneurons are similar. Synaptic activation is described by 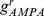, 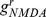 and 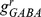, obeying the dynamics
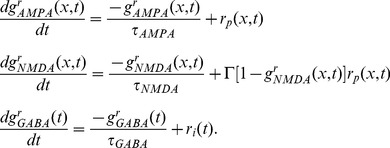
(17)Because the NMDAR time constant is much longer than the respective time constants of AMPARs, GABARs and neuronal firing rates (Equations 13 and 14), these last three variables can be given their steady state values, while NMDAR activation dominates the dynamics of the system. Thus, the system can be described by
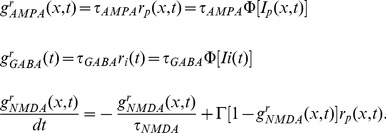
(18)By further linearizing the activation function of the interneurons 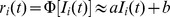, we obtain the integral and partial equation to approximate the timing network:
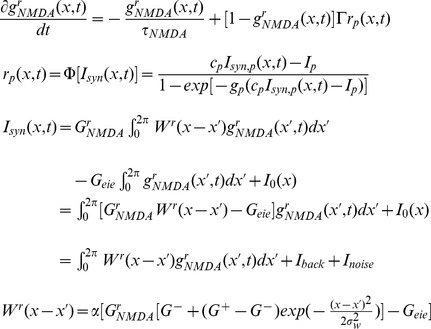
(19)The chosen parameters were 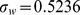 (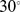), 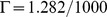, 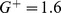, 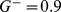, 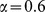, 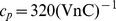, 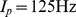, 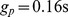, 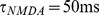, 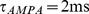, 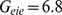, 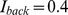. Scale factors 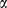, 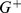 and 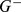 were used to tune the model to qualitatively reproduce the steady state firing rates in the timing network, while 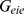 abstracts over the unstructured interactions between pyramidal neurons and interneurons.

## Results

We used the generic local-circuit model to simulate an interval estimation task, where intervals were estimated by the time that climbing activity crossed a fixed threshold. We coupled two instances of the local-circuit model to simulate a decision task, where the timing network governed the rate at which the decision network distinguished between two stimuli. As described above, the decision network was identical to the timing network, including all parameter values except for NMDAR conductance strength. Our results proceed as follows: (1) we show that climbing activity in the timing network is controlled by the strength of NMDAR conductance, encoding intervals by the time it takes to reach a fixed threshold. We explain the mechanism underlying this phenomenon by dynamic analysis of the reduced system. (2) We show that the interval estimates by the timing network conform to the scalar property of interval timing, a widely observed experimental phenomenon in which the standard deviation of interval estimates scales linearly with the mean [13], [55]. We explain how the scalar property emerges from the timing network by deriving the probability density of first passage times of the timing threshold. (3) We show that a simple learning rule is sufficient for the timing network to learn to estimate different intervals in the hundreds of milliseconds range. (4) We demonstrate several biologically plausible mechanisms for starting and stopping interval estimates by the timing network, each of which qualitatively reproduces electrophysiological data from tasks with a timing requirement. (5) We demonstrate that the timing network controls the SAT in the downstream decision network, using a biologically plausible means of gain modulation. (6) We show that the resulting distribution of decision times reproduces signature characteristics of decision times by experimental subjects.

### Interval estimates are controlled by NMDAR conductance strength in the timing network

We simulated an interval estimation task with the generic local-circuit model. Estimates of different intervals were produced by scaling the conductance strength of NMDARs by 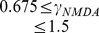, where different values of 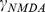 supported different rates of buildup of activity by the bump population. On each trial, the time at which the mean SDF of the bump population reached a threshold of 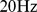 was considered the interval estimate. Thus, like earlier authors (*e.g.* [56], [57]), we assume that a behaviourally relevant ballistic process is initiated downstream when neural activity encoding the interval estimate reaches a certain firing rate. The task was simulated by running the network for 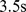, sufficient time for a bump to develop for all of the above values of 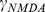 on at least 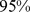 of trials. This length of time may seem long for estimates in the hundreds of milliseconds range, but for longer interval estimates, it allowed for the growing variability of estimates with interval duration, commonly seen in interval estimates in experimental tasks (see Results section *The scalar property of interval timing*). For 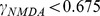, bumps did not consistently develop within the allotted time, and for those that did, spiking activity did not consistently reach 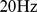. For 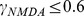, background spiking was approximately 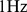 among pyramidal neurons and 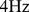 among interneurons [58], but climbing activity was not supported by the network. An upper limit of 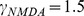 was used because it is consistent with the experimental enhancement of the NMDAR component of cortical excitatory post-synaptic currents by approximately 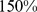 of baseline [59], [60] and because interval estimates were increasingly indistinguishable above this value. 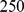 trials were run for each value of 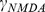.

**Figure 2:**
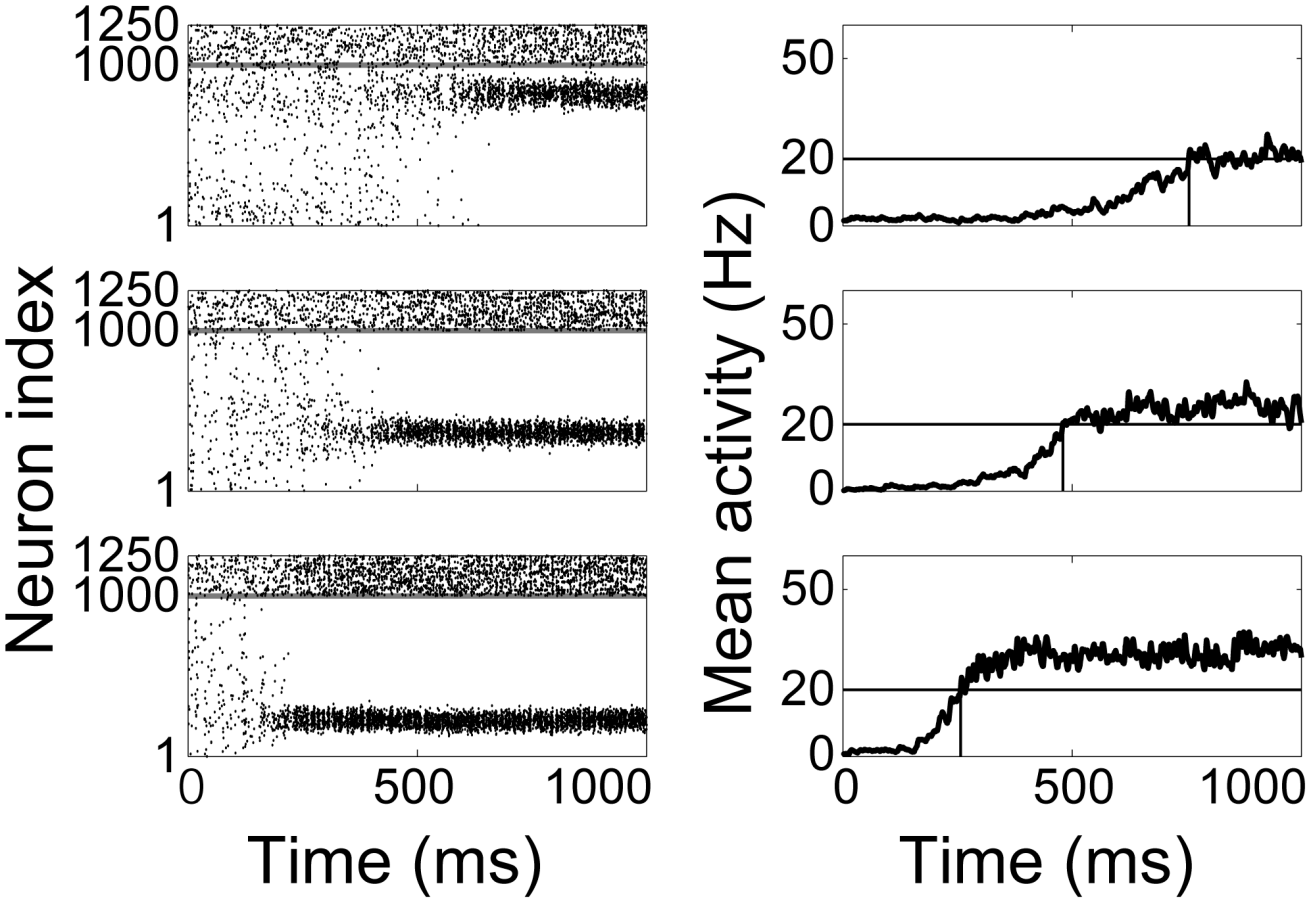
Raster plots (left) and mean SDFs over the ‘bump’ population (right) show the timing network coding temporal intervals with 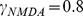 (top), 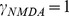 (middle) and 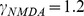 (bottom). Intervals were estimated when the mean SDF of the bump population reached 20 Hz. In raster plots, pyramidal neurons and inhibitory interneurons are indexed from 1–1000 and 1001–1250 respectively, indicated by the horizontal grey line.

Varying the scale factor 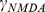 furnished a range of rates of buildup activity, where lower values of 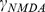 lead to slower buildup and higher values lead to faster buildup. The lowest value of 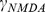 that consistently supported buildup activity produced a mean interval estimate of 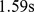 (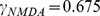). The highest value of 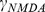 consistent with experimental enhancement of cortical EPSCs [59], [60] produced a mean estimate of 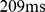 (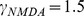). The timing network thus supported interval estimates from approximately 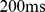 to 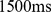, consistent with experimental evidence that temporal coding on this order is supported by a common mechanism [12], [55]. Example trials for three values of 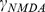 are shown in Figure 2. Note that the location of the bump differs on each trial, as there is no bias favouring a particular network location. We are unaware of any data to conclusively confirm or refute such trial-to-trial variability, but to produce climbing activity in the same sub-population from trial to trial, we simply need to strengthen excitatory synaptic conductances among a few localized neurons, *e.g.* by Hebbian long term potentiation among the neurons participating in the bump. In the coupled-circuit decision trials in Section *Encoding time constraints for a decision*, the location of climbing activity in the timing network does not matter because projections from the timing network to the decision network are spatially non-selective.

**Figure 3:**
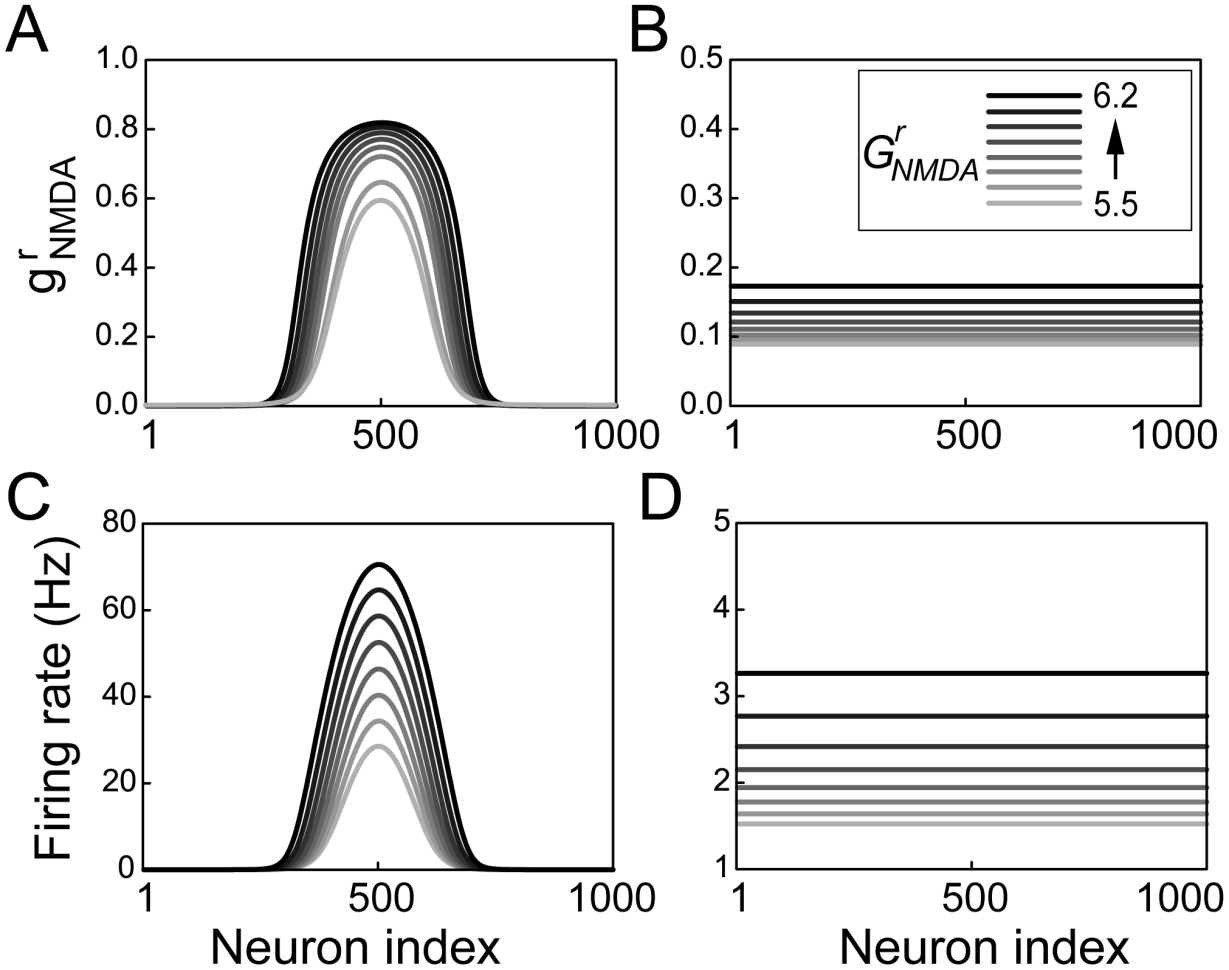
Two steady states of the reduced system. (A) NMDAR activation at the stable bump state for synaptic strengths 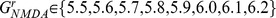. Darker curves correspond to higher values of 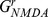 (see legend in panel B). (B) NMDAR activation at the unstable steady state. (C) Firing rates at the stable steady state. (D) Firing rates at the unstable steady state. Shades of grey in B, C and D correspond to those in A.

The mechanism underlying climbing activity in the timing network can be understood by non-linear analysis of the reduced integral and partial differential system. For a given value of the effective synaptic strength 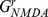, corresponding to NMDAR conductance strength in the timing network, the steady states of the reduced system can be calculated by setting the right hand side of Equation 19 to zero and solving the resulting equations. Our analysis revealed three regimes of the reduced system. 1) Sufficiently small values of 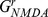 furnished a flat steady state which is stable and whose eigenvalues are negative. This regime corresponds to the common case in cortex, where background activity is stable and stimulus-selective activity decays to this background state after stimulus offset. This regime in the reduced system corresponds to approximately 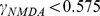 in the timing network. 2) With a moderate increase in 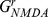, the system enters a bistable regime. The stable flat steady state is retained, but a small unstable bump steady state and a large stable bump steady state emerge. This bistable regime corresponds to the classic persistent storage regime in these networks (*e.g.* [21], [26]), in which a stimulus can trigger a bump state, which persists after stimulus offset. This regime in the reduced system corresponds to approximately 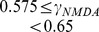 in the timing network. 3) With a further increase in 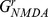, the stable flat state and the unstable bump steady state coalesce into one unstable flat steady state whose largest eigenvalue is positive, while the stable bump state becomes higher. The magnitudes of the unstable flat steady state and the stable bump state increase with further increase to 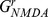. This regime in the reduced system corresponds to approximately 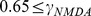 in the timing network. This third regime is shown in Figure 3, where panels A and B show NMDAR activation at the stable bump state and the unstable flat steady state respectively with increasing 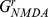. Panels C and D show the corresponding firing rates 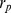.

**Figure 4:**
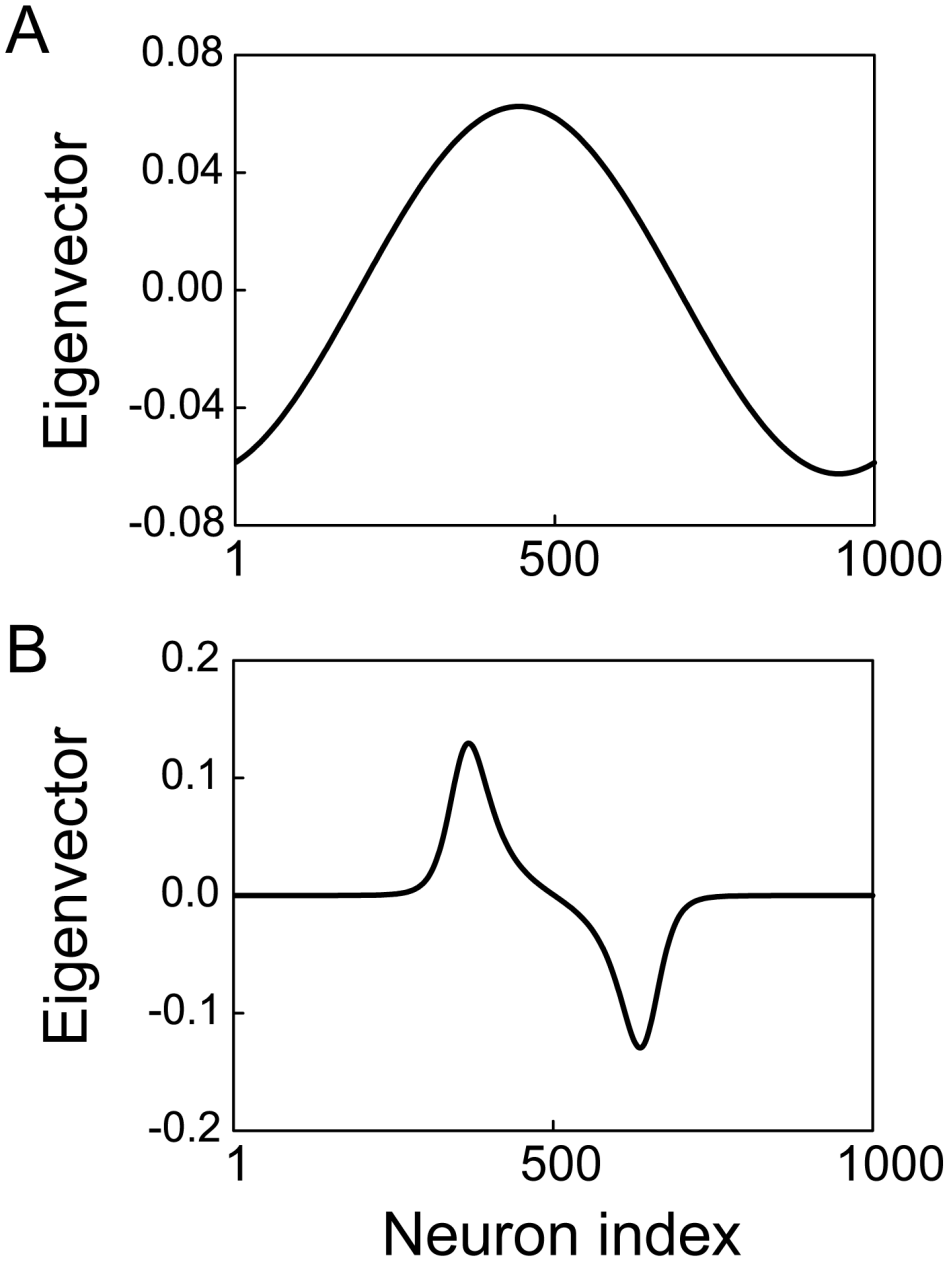
Eigenvectors of the reduced system. The first eigenvector of the unstable flat steady state (A) and stable bump steady state (B).

The instantaneous firing rates 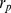 of the stable bump states in the reduced system were consistent with the steady state spike rates in the timing network, ranging from approximately 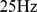 to 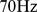 as 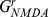 was increased from 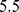 to 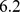, corresponding to an increase in 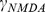 from 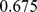 to 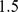 in the timing network (Figure 3C). The evolution of the system away from the unstable flat steady state is dominated by the largest positive eigenvalue and a localized activity bump emerges due to the corresponding eigenvector (Figure 4). On the other hand, the largest eigenvalue of the stable bump steady state is zero and the corresponding eigenvector explains the invariant location of the bump [61]. Climbing activity therefore occurs at arbitrary locations, as shown in Figure 2 above.

### The scalar property of interval timing

**Figure 5:**
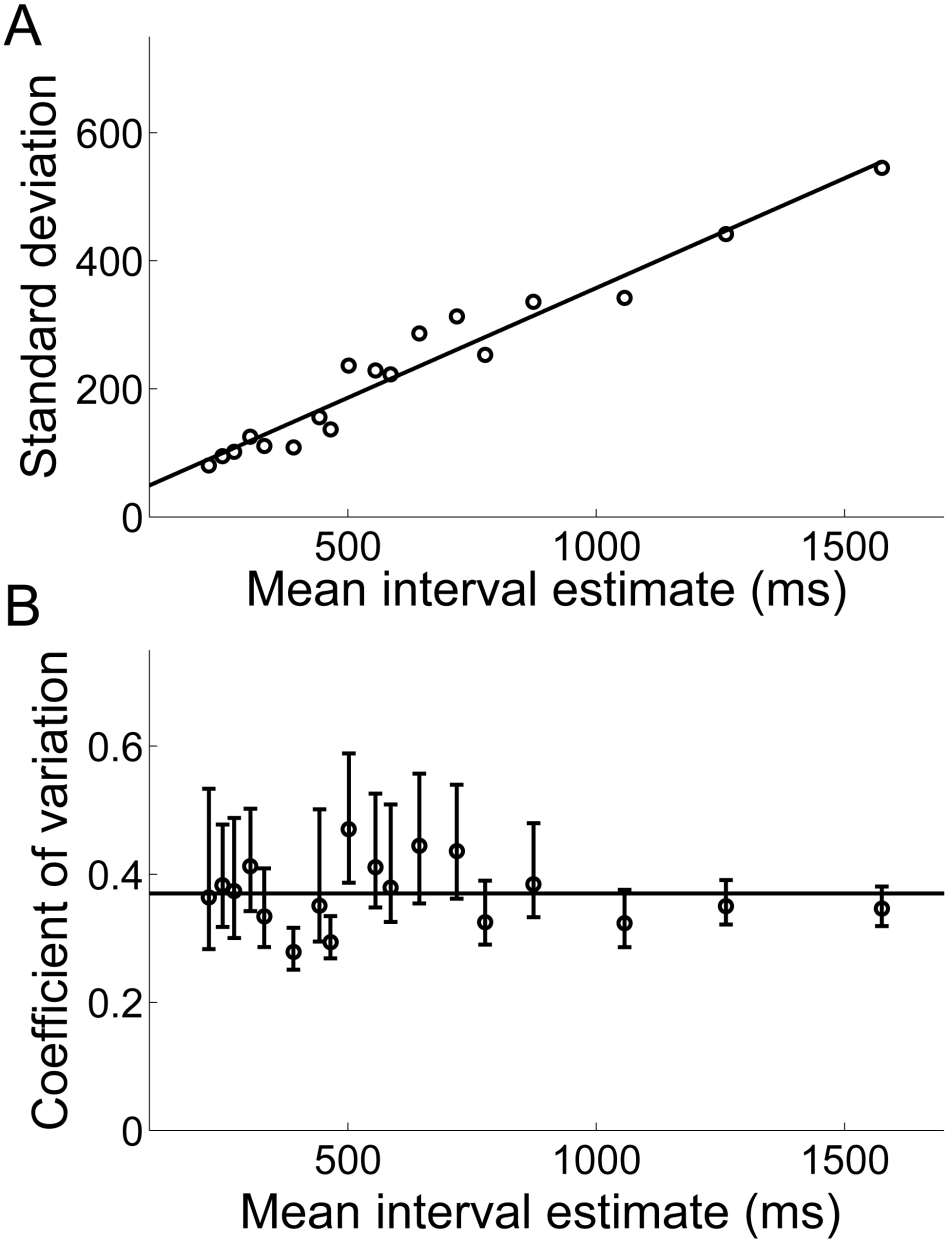
The interval estimates produced by the timing network conform to the scalar property of interval timing. (A) The standard deviation over the mean interval produced by the timing network for each value of 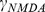. The plotted line shows the best linear fit (least squares). (B) Coefficient of variation (CV) over the mean interval for each value of 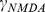. Error bars show 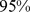 confidence intervals. The plotted line shows the best horizontal linear fit. As the estimates increase in length, the data converge to the slope of the linear fit in A.

**Figure 6:**
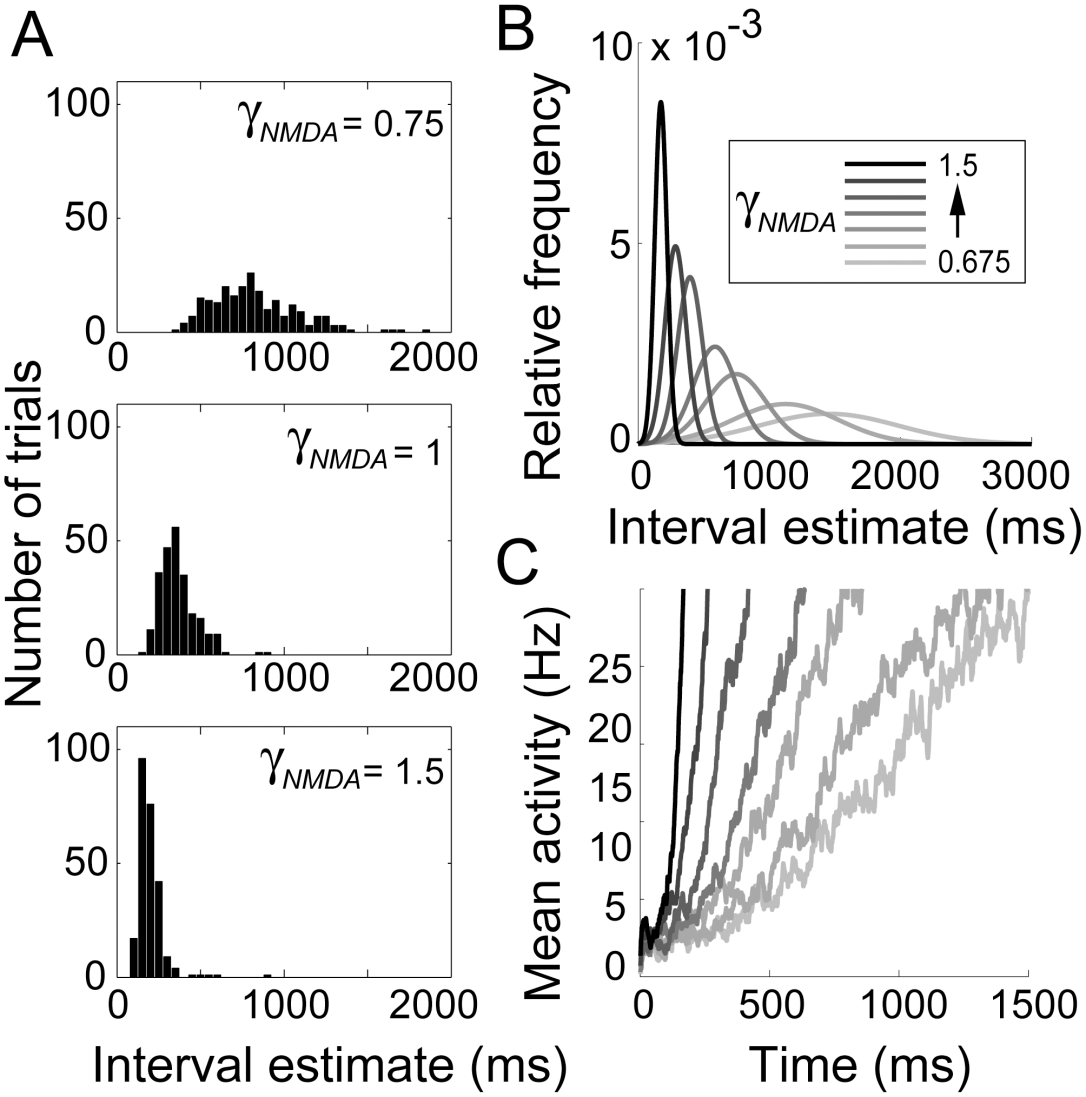
Interval estimates produced by the timing network. (A) Distribution of intervals produced by the timing network for 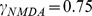 (top), 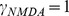 (middle) and 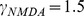 (bottom). Histogram bins were 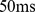. (B) Gaussian fits to the distribution of intervals produced for 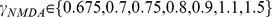, the first and last of which correspond to the lowest and highest values of 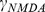 (see Methods). (C) Mean SDFs at the centre of the bump (see Methods) over all trials for the values of 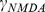 shown in *B*.

Not only did the timing network produce interval estimates, but the estimates conformed to the scalar property of interval timing [62]. The scalar property is a strong form of Weber's law where the standard deviation of estimates is proportional to the mean (Figure 5A). Weber's law is widely regarded as a signature characteristic of interval timing across a wide range of temporal orders [13], [55], though see [63] and [64] for a systematic description of conformities and violations of the scalar property in humans and non-human animals respectively. The coefficient of variation (CV, the standard deviation divided by the mean) of the interval estimates produced by the timing network was approximately constant (Figure 5B) and compared favourably to experimental measurements on this order [55], [65]. The distribution of interval estimates for each value of 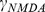 was roughly normal (Figure 6A), another widely-reported characteristic of interval estimates across a range of temporal orders [13], [55]. Gaussian fits to the estimates are shown in Figure 6B. For comparisons with experimental data in the hundreds of milliseconds range, see *e.g.* [66] and [67].

Climbing activity in the timing network can be understood as the evolution of the system from an initial state in the vicinity of the unstable flat steady state to the stable bump state. We linearized the system in the vicinity of the unstable flat steady state as 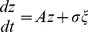, where 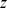 denotes 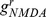 for simplicity. To consider the effects of noise, the linear system can be expressed as a Langevin equation 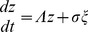, where 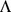 is the eigenvalue of the matrix 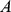, 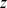 is a vector, and 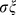 is white noise with standard deviation 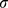. According to non-linear dynamics, the system expands along the manifold tangent to the eigenvector with positive eigenvalue. Thus, we focus on the largest positive eigenvalue, which dominates the expansion of the system [68], and further simplify the system as a 1-dimensional Ornstein-Uhlenbeck (OU) process
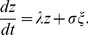
(20)The parameter 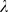 of an OU process is typically negative, supporting a stable distribution. Here, 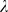 is positive because the flat steady state is unstable. Thus, positive 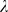 implies that the system departs from the flat state starting at initial state 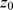. The corresponding Fokker-Plank equation can be written as
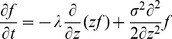
(21)with initial state 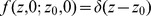. The distribution of arrival times of 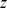 at the timing threshold can be calculated as
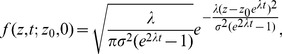
(22)which shows that the system grows along the curve 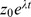 with standard deviation 
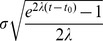
.

Intervals are estimated when the system reaches the threshold 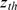, so the interval estimates are the first passage times of the OU process, the distribution of which can be calculated as
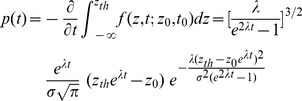
(23)with mean 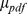 and variance 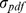 given by
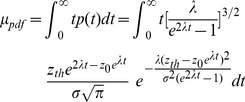
(24)and
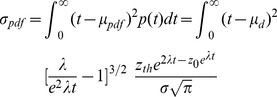
(25)respectively.

Of note, 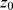 and 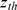 are the initial value and threshold of NMDAR activation, (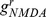, Equation 19), not the firing rate. To calculate the distribution of first passage times numerically, we need to express the threshold in terms of 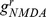. For each value of 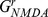, we therefore calculated 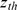 by scaling the interval estimation threshold in the timing network by the ratio of the maximum values of 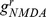 and 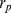, *i.e.* 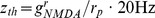. This scaling preserves our use of a fixed firing rate threshold in the timing network. Values for 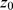, 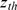 and the largest positive eigenvalue 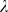 are given in Table 1 for increasing 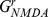. We used a constant level of background noise for all simulations (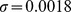), consistent with our use of constant parameters with the background noise (point-conductance) model across the different values of 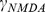 in the timing network.

**Figure 7:**
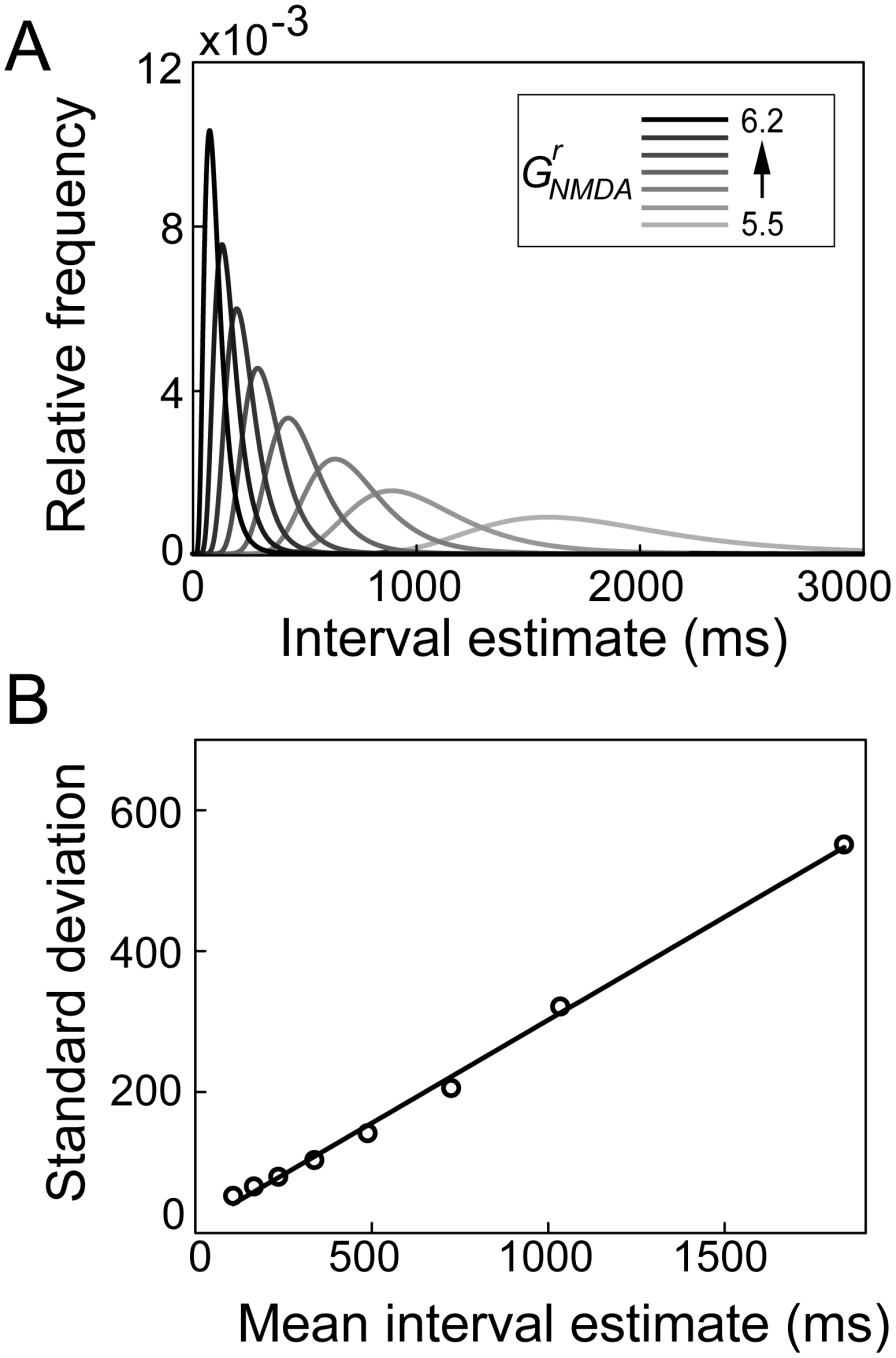
The probability density of first passage of the timing threshold by the reduced system. (A) Probability density of interval estimates for different values of NMDAR conductance strength 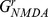 in the reduced system. (B) The standard deviation over the mean for curves shown in A.

The distribution of first passage times is shown in Figure 7. These curves are very similar to the distribution of interval estimates by the timing network (compare Figures 6B and 7A). Stronger (weaker) NMDAR conductance causes faster (slower) ramping and a narrower (wider) distribution, while the relationship between the mean and standard deviation is approximately linear (compare Figures 5A and 7B).

### Learning interval estimates

**Figure 8:**
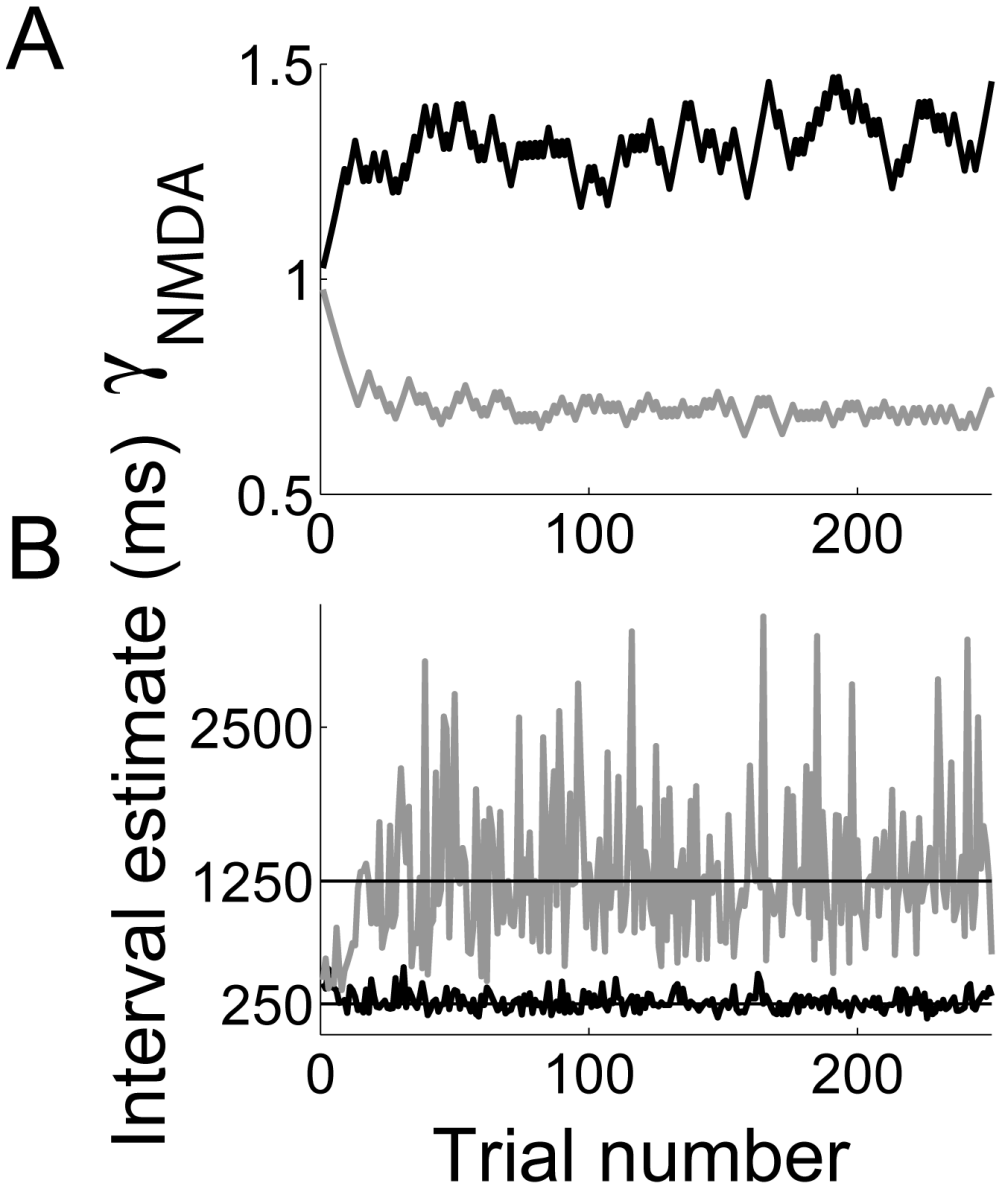
The timing network learns to estimate intervals under the rule given by Equation 26. Black and grey curves correspond to specified intervals of 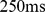 and 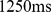 respectively. (A) Trial-to-trial fluctuations of 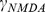 during learning. (B) Trial-to-trial interval estimates.

The previous sections demonstrate that the timing network estimates intervals in the hundreds of milliseconds range as a function of the scale factor 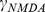 and that these estimates share signature characteristics with those of experimental subjects in studies of interval timing. Next, we consider whether the network can learn a given interval in this range, using a simple learning rule [57]. We ran the interval estimation task (described above in Results section *Interval estimates are controlled by NMDAR conductance strength in the timing network*) for desired intervals 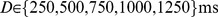. For each desired interval, the network began the block of trials in the baseline condition (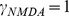) and 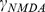 was adjusted after each trial 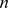 according to
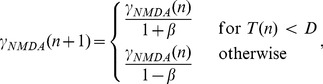
(26)where 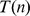 is the estimate of 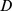 on trial 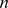 and 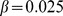 determines the rate of learning. As shown in Figure 8, the network learned each interval after a handful of trials [7], [32].

### Starting and stopping the estimate

**Figure 9:**
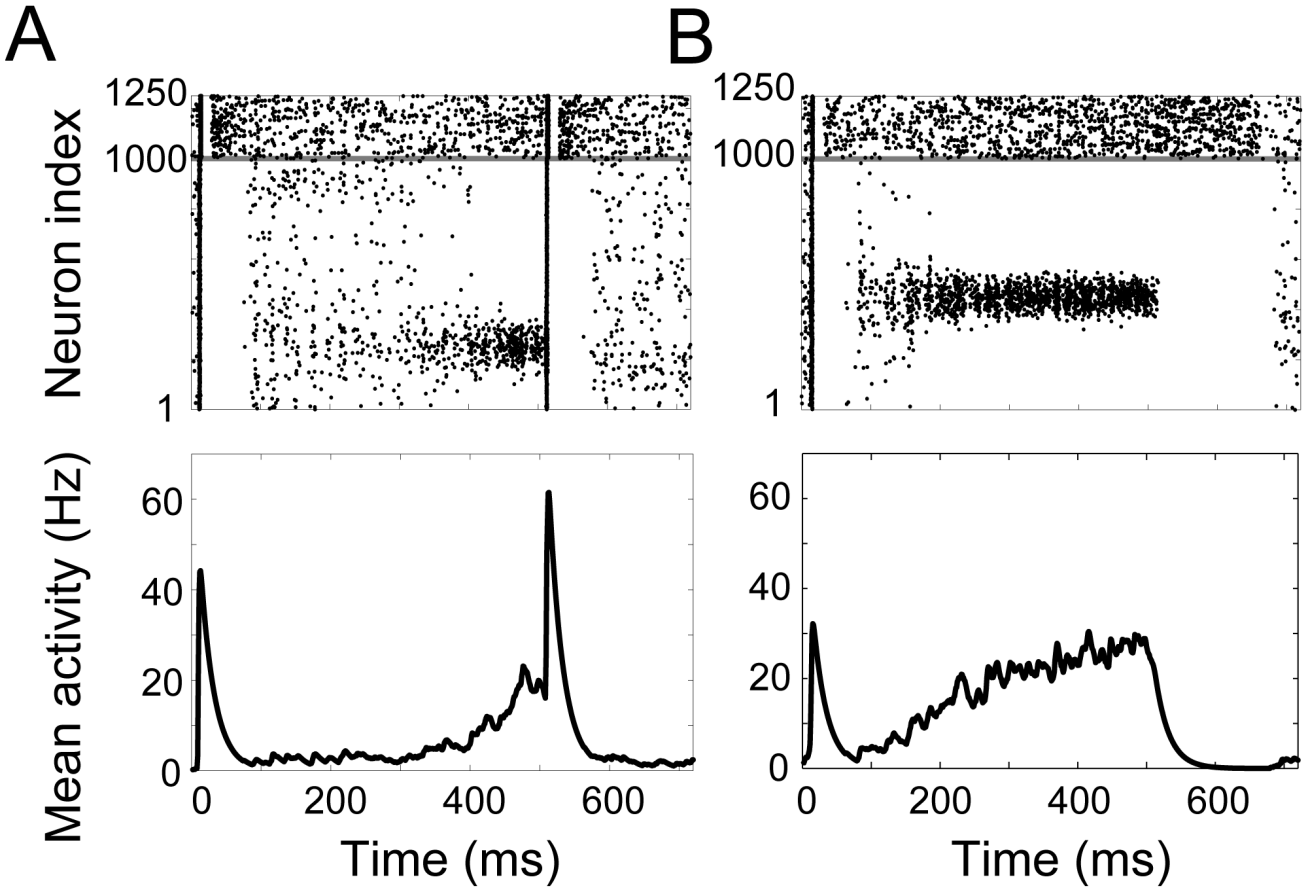
Starting and stopping the timing network. In raster plots (upper), pyramidal neurons and inhibitory interneurons are indexed from 1–1000 and 1001–1250 respectively, indicted by the horizontal grey line. Lower plots show mean SDF of the bump population for the same simulations. See text for simulation details. (A) Brief, spatially non-selective excitation of all pyramidal neurons resets the network, regardless of its current state. (B) Brief, spatially non-selective excitation and inhibition of pyramidal neurons starts and stops activity in the timing network respectively.

Estimates of elapsed time occur relative to a start time, so the network requires a start signal to begin each estimate. Such a signal should be able to reset the network to the background state, regardless of its current state. There are a number of plausible mechanisms that could play this role. We demonstrate two such mechanisms. One is a brief pulse of spatially non-selective excitation, generating blanket feedback inhibition and thus shutting the network down. This mechanism was demonstrated in an earlier study simulating persistent mnemonic activity in prefrontal cortex, using a local-circuit model similar to ours [21]. Figure 9A shows this mechanism in the timing network, where the average excitatory conductance of the point conductance model (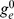) at pyramidal neurons was increased by a factor of 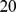 at all pyramidal neurons for 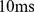 to start the estimate, and again at time 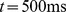 to stop the estimate. In this case, the start signal potentially corresponds to broad excitation of the timing network by a cue stimulus, while the stop signal potentially corresponds to efference copy at the time of motor initiation [22]. As such, we do not expect these signals to be identical in duration or magnitude, but giving them the same parameters shows robustness of the mechanism (fine tuning of each signal was not necessary). Electrophysiological data showing a similar trajectory can be seen in *e.g.* [69], where these data were interpreted as encoding the anticipation of an upcoming stimulus.

Another plausible reset mechanism is long-range excitatory targeting of inhibitory interneurons, which in turn inhibit local pyramidal neurons [70]. Such disynaptic inhibition has been suggested to underlie the control of motor initiation in anti-saccade [71] and countermanding tasks [72] and is simulated in the timing network in Figure 9B. In this simulation, the average excitatory conductance of the point conductance model (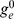) at pyramidal neurons was increased by a factor of 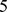 for 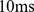 to start the estimate, and the average excitatory conductance at interneurons was increased by a factor of 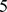 for 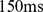 at 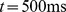 to stop the estimate. Similar electrophysiological data can be seen in *e.g.* [73], interpreted as encoding the anticipation of an upcoming stimulus in their study. There are, of course, other mechanisms that could start and stop estimates of elapsed time by climbing activity. Indeed, we do not expect cortical timing circuits to remain indefinitely in a regime with no stable background state. For example, at the onset of a cue stimulus, fast-acting neuromodulation could alter network dynamics in a manner similar to the scaling of 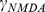, or cortico-thalamo-cortical disinhibition could have a similar effect.

### Encoding time constraints for a decision

**Figure 10:**
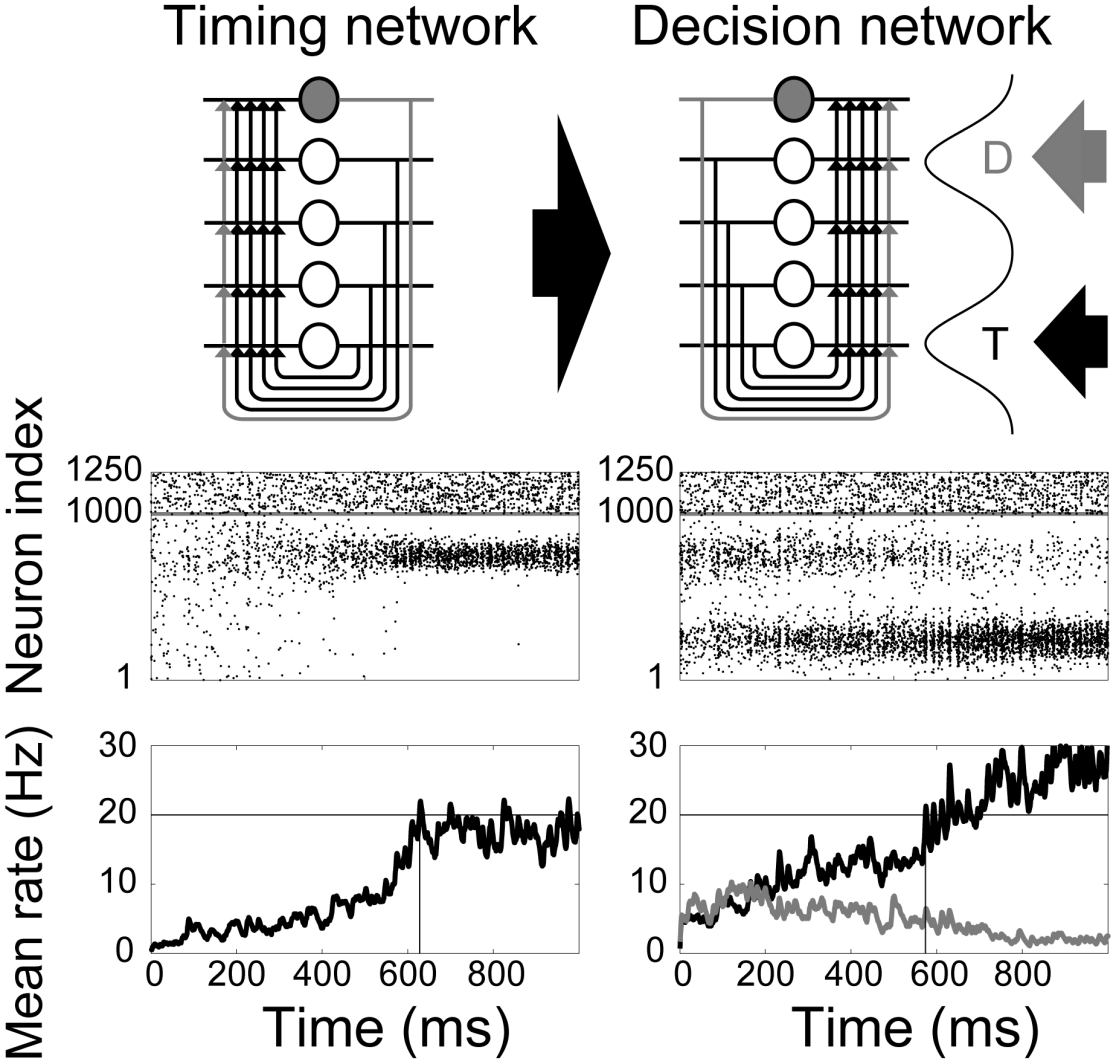
Gain modulation of the decision network (right) by the timing network (left). (Top row) Each network schematic reproduces the schematic in Figure 1A. Climbing activity in the timing network provides spatially non-selective input to the decision network, depicted by the broad arrow. The decision network has Gaussian response fields for the target (T) and distractor (D) stimuli (see Methods), where the thinner arrows depict incoming evidence. (Middle row) Raster plots for each network during a single trial of the decision task with 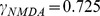 and task difficulty 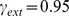. Pyramidal neurons and inhibitory interneurons are indexed from 1–1000 and 1001–1250 respectively, indicated by the horizontal grey line. Neurons 250 and 750 are the centres of the target and distractor RFs respectively. (Bottom row) Mean SDFs over the bump population in the timing network and the target and distractor populations in the decision network (see Methods). Black horizontal lines indicate the 20 Hz threshold used for interval estimation and decision making in the respective networks. Vertical lines show the time of threshold-crossing.

To address the hypothesis that the encoding of elapsed time controls the speed and accuracy of decisions by gain modulation [11], we ran further simulations to determine if the timing network's temporal estimates could control the SAT in a downstream network during a decision task (Figure 10). As indicated above, the two networks were identical except for the inputs they received and the scale factor 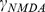. To emphasize the role played by the timing network in these simulations, 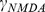 was given a low value in the decision network (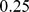, one quarter of the baseline NMDAR conductance in the timing network for synapses onto pyramidal neurons and interneurons) so its intrinsic processing was too weak to make decisions across all task difficulties without spatially non-selective input from the timing network. Note that this low value of 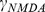 did not support climbing activity in the absence of selective input. Down-scaling NMDARs was thus a practical means of limiting the decision network's processing capability. Although we do not assign it a specific biological correlate, we note that the properties of NMDARs can show marked variation between cortical regions [74] and under receptor modulation within a single region [60], [75].

We simulated a two-choice decision task by providing two noisy stimuli to the decision network for 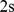. On each trial, the network's task was to distinguish the higher-rate input (the target) from the lower-rate input (the distractor). For each stimulus, independent, homogeneous Poisson spike trains were provided to all pyramidal neurons in the decision network, where spike rates were drawn from a normal distribution with mean 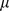 corresponding to the centre of a Gaussian response field defined by 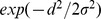. Constants 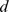 and 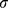 are given above for the pyramidal interaction structure 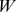 (Methods section *The network model*). For the target stimulus, we simulated 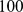 upstream stimulus-selective neurons firing at 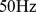 each by setting 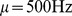 and setting extrinsic AMPAR and NMDAR conductance strength to 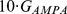 and 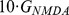 respectively, trading spatial summation for temporal summation [76]. The distractor stimulus was similarly defined, where task difficulty (target-distractor similarity) was determined by multiplying 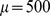 by 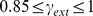. On each trial, the decision was considered correct (incorrect) when the mean SDF of the target-selective (distractor-selective) population reached a threshold of 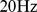. As with the interval estimation task, the threshold assumes a downstream ballistic process is initiated when neural firing reaches a certain rate, an assumption supported by a large body of experimental and theoretical work from decision tasks (see [1], [3], [77], [78]. The time of threshold-crossing was considered the decision time. Note that the precise value of the threshold was not crucial.

Gain modulation of the decision network by the timing network was implemented by spatially non-selective excitation [24], [33], that is, each pyramidal neuron in the decision network received input from all pyramidal neurons in the timing network for the entirety of each trial. Only AMPAR conductances mediated these inter-network inputs, which were set to one fifth the strength of extrinsic AMPARs.

The total input current to each neuron 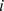 in the decision network was therefore
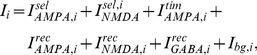
(27)where 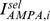 and 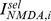 mediate stimulus-selective inputs (set to 0 for interneurons), and 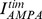 mediates spatially non-selective inputs from the timing network (set to 0 for interneurons). NMDAR and AMPAR activation at these synapses follows Equations 5 and 6 above.

**Figure 11:**
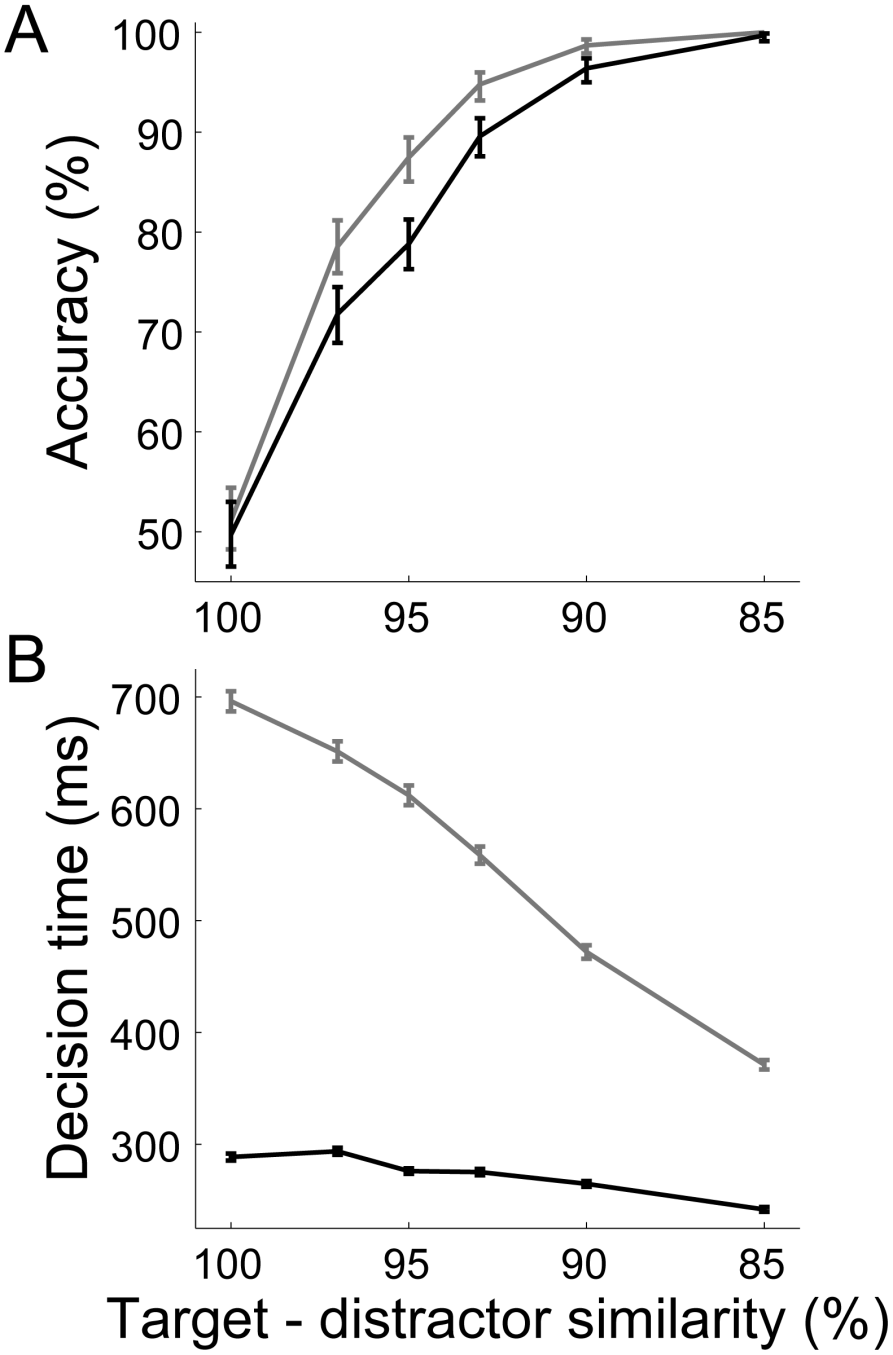
Temporal estimates by the timing network control the speed-accuracy trade-off in the decision network. Black and grey curves show results for temporal estimates of 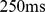 and 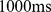 respectively. (**A**) Accuracy: the proportion of trials on which the decision network correctly chose the target for each level of task difficulty (target-distractor similarity). Error bars show 95% confidence intervals. (**B**) Decision time: mean time of threshold-crossing (20 Hz) by the target or distractor population (see Methods). Error bars show standard error.

A block of 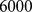 decision trials (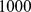 trials for 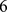 task difficulties) was run for values of 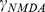 learned by the timing network for a short (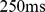) and a long (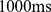) interval (Section *Learning interval estimates* above). The mean value of 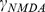 over the last 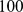 trials was used in each case. Tight and loose temporal constraints were thus imposed on the decision task by running the timing network with 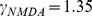 and 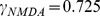 respectively on each block of trials, where activity in the timing network served as a spatially non-selective input to the downstream decision network. In both temporal conditions, the model was very accurate on the easiest task (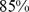 mean target-distractor similarity) and performed at chance when the inputs were indistinguishable on average (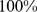 mean target-distractor similarity). For task difficulties in between, decisions were more accurate with the longer temporal estimate. Decision times were shorter for all task difficulties with the shorter temporal estimate. The coupled-circuit decision model thus traded speed and accuracy as a function of a learned interval (Figure 11).

#### Distributions of decision times under speed and accuracy conditions

Just as the the timing network reproduced signature characteristics of interval estimates by experimental subjects, the coupled-circuit decision network reproduced signature characteristics of psychophysical data from decision tasks. As described in [77], these characteristics result from within-block and between-block experimental manipulations. Our within-block manipulation is task difficulty (mean target-distractor similarity) and our between-block manipulation is the imposition of speed and accuracy conditions by short (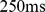) and long (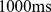) interval estimates respectively.

**Figure 12:**
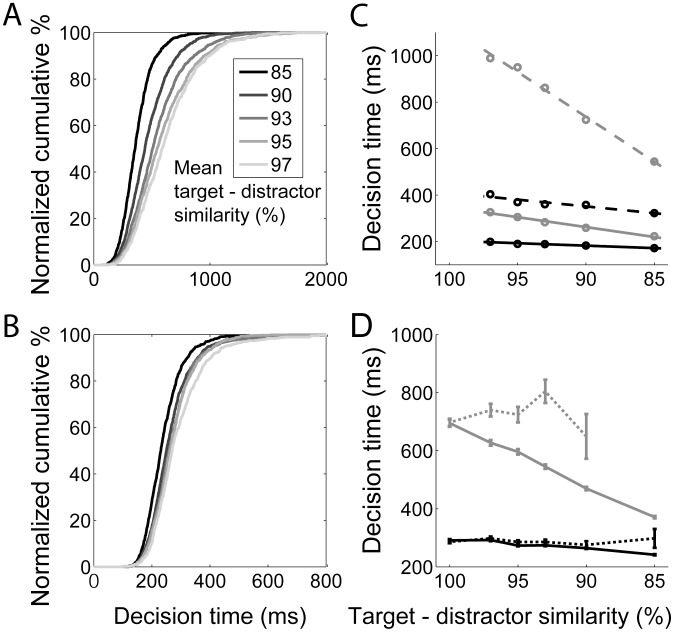
Distributions of decision times produced by the coupled-circuit model. (A) Normalized cumulative distributions for correct trials under accuracy conditions. Darker curves correspond to more difficult tasks, determined by mean target-distractor similarity (see legend). (B) Same as in *A*, but under speed conditions. Note the different scales of the x-axis in A and B. (C) Decision times at the crossing of quantiles 0.1 and 0.9 of the distributions for correct trials under accuracy (grey) and speed (black) conditions, plotted as a function of task difficulty. Curves show the best least squares linear fit, where solid and dashed lines correspond to quantiles 0.1 and 0.9 respectively. Under each condition, the slope of the dashed line (38.6 grey, 5.8 black) is greater than that of the solid line (8.5 grey, 2.1 black), indicating that the tail of the distribution is growing more than the leading edge as difficulty increases. (D) Mean decision times on correct (solid) and error (dotted) trials under accuracy (grey) and speed (black) conditions. Error bars show standard error.

Three general within-block findings are identified in [2], [77]: (1) accuracy decreases and reaction times (RT) increase on correct trials with increasing task difficulty; (2) the shapes of RT distributions are positively skewed, where on correct trials, the tail of the distribution grows at a greater rate than the leading edge with increasing task difficulty; and (3) mean RT differs on correct and error trials, depending on conditions. All three general findings are reproduced by the model (Figures 11 and 12), where we ignore the non-decision component of RT, known to have minimal effect on RT distributions [79]. As described above, increasing task difficulty resulted in a decrease in accuracy and an increase in mean decision time (DT) under both between-block conditions. Increasing mean target-distractor similarity from 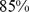 to 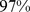 resulted in a decrease in accuracy from 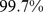 to 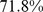 and an increase in mean DT from 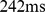 to 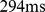 under speed conditions; and resulted in a decrease in accuracy from 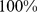 to 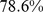 and an increase in mean DT from 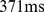 to 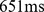 under accuracy conditions (Figure 11). Furthermore, under both between-block conditions, the shapes of DT distributions were positively skewed (Figure 12A,B) for all task difficulties (not shown), the tail of the DT distribution for correct trials grew at a greater rate than the leading edge with increasing task difficulty (Figure 12C, see caption), and error trials were systematically longer than correct trials (Figure 12D).

## Discussion

Over the past several decades, a wealth of data and theory have characterized the cortical mechanisms underlying decisions for action in the world, such as where in space to foveate or to reach (see [1], [3]). In this characterization, time is a passive vehicle for the filtering of noisy spatial codes (see [4]). Recently, there has been increasingly broad recognition that the encoding of time is an essential determinant of behaviour [12], [13], [32], [55], [80], [81]. It would, after all, be impossible to plan actions or anticipate upcoming events without a representation of time. Psychophysical studies have characterized temporal coding for decades [62], [67], [82], [83] and a growing body of electrophysiological [73], [84]–[89], neuroimaging [90], [91], and theoretical [56], [57], [92]–[96] studies have begun to reveal the neural mechanisms underlying the representation of time. Despite these developments, very few studies have considered the interactions between spatial and temporal codes for perception, decision and action [8]–[11].

Using a generic, local-circuit cortical model, we have proposed a neural mechanism for the encoding of time in the hundreds of milliseconds range. Climbing activity in the model resembles neural activity in a number of cortical regions during experimental tasks with a timing requirement in this range [9], [69], [73], [84]–[89] and our analysis of network dynamics formally characterizes the underlying mechanism. Strong feedback excitation destabilizes the network's background state, repelling the network toward a stable attractor. Because the dynamics evolve more quickly with stronger feedback excitation, estimates are shorter with stronger intrinsic NMDAR conductance. The model's interval estimates conform to Weber's law, widely observed experimentally (Figures 6 and 5) and the CV of the these estimates is consistent with that of experimental subjects in timing tasks in the same range (Figure 5). Our derivation of the probability density of interval estimates reveals Weber's law as a mathematical property of the proposed timing mechanism (Figure 7). A simple learning rule is sufficient for the network to learn new intervals within a handful of trials (Figure 8), as observed across experimental paradigms and species [7], and we have demonstrated plausible mechanisms for starting and stopping the estimates (Figure 9). Consistent with the hypothesis that generic properties of local cortical circuits support spatial and temporal processing [12], an identical downstream network makes decisions in simulations of a perceptual discrimination task, producing psychometric and chronometric curves consistent with experimental data (*e.g.* [9], [97], [98]) and demonstrating the SAT as a function of the learned temporal estimates upstream (Figure 11).

### The representation of time in the hundreds of milliseconds range

Different neural mechanisms are expected to code for widely varying temporal durations, ranging from microseconds to days [12], [13]. While considerable overlap between mechanisms is expected at timescales in between, it has been proposed that a dedicated mechanism exists for the hundreds of milliseconds range (see [55], [67]), the relevant order for the most well-studied perceptual decision tasks [1], [3], [14]. These proposals are based on the premise that a single mechanism encoding time for different tasks and modalities will show common variability in these different contexts. For example, [55] suggested a dedicated timing mechanism in this range based on pooled data from a variety of tasks and species showing a similar CV from approximately 200 to 1500 ms (much like Figure 5B). Along similar lines, [82] described a constant Weber fraction for 200 to 2000 ms. [83] used the slope analysis method to distinguish timing-based variability from non-timing sources of variability, such as variability due to perceptual and motor processing during timing tasks. Under this approach, the slope of the linear fit to the variance plotted over the square of interval durations reveals the time-dependent process, shown in their study to be similar for intervals from 325 to 550 ms in two timing tasks. [67] showed a common slope under this method for several auditory tasks requiring interval estimates from 350 ms to 1 s, though they also showed significantly different slopes for other auditory tasks, visual tasks, and between auditory and visual implementations of the same task. See their study for a more extensive description of the evidence for and against a common timer in this range.

In consideration of the above, it is important to distinguish between a common mechanism for timing and a common timer. A common timer refers to a ‘central clock’ processing time across a set of modalities and tasks. Inherent in this definition is a common mechanism, but a common mechanism does not necessarily imply a common timer. We propose that the capability to code time in the hundreds of milliseconds range is a generic property of local cortical circuits under conditions supporting strong attractor dynamics, but this capability does not imply that any single circuit should code time for all tasks and modalities, nor that all local cortical circuits should code time.

Our model fits a distributed processing framework, with local circuits coding time in various cortical regions across different tasks and modalities, supported by inherent properties of local-circuit cortical processing [95], [99]. There is considerable debate about the strength of evidence supporting distributed vs. central timers for different temporal durations, modalities and tasks (see [15], [17], [90], [100]), but the growing volume of neural data showing climbing activity in the hundreds of milliseconds range in different cortical regions during tasks with a timing requirement provides strong support for a distributed framework. For example, climbing activity in this range has been recorded in several regions of prefrontal cortex, including lateral [69], [73], anterior cingulate [84], [101] and premotor [85] regions in anticipation of upcoming events; as well as in parietal areas 7A [86] and LIP [9], [87], [102]. Similar activity has been recorded in anticipation of reward in primary visual cortex [88], in the timing of movements in the absence of environmental cues in LIP [89], and in the midbrain superior colliculus during predictable delays [103]. Furthermore, many of these data showed a phasic response at the start of the anticipatory periods, suggesting a reset mechanism (Figure 9) that would allow for the encoding of elapsed time relative to a start time (*e.g.* [69], [73], [88], [89]). It is important to note, however, that in a hierarchical cortical framework, one or several local circuits could conceivably encode time at the top of the hierarchy for use in more peripheral processing, *i.e.* despite favouring a distributed framework, we acknowledge that our model could also support a centralized framework.

### Prospective and retrospective coding

We have focused on the representation of time in the hundreds of milliseconds range by climbing activity, but climbing activity is not limited to the hundreds of milliseconds range, nor is climbing activity the only neural data indicative of temporal coding on this order. Such activity is generally regarded as ‘prospective’ coding [73], [104], *i.e.* neural activity encoding elapsed time in anticipation of an upcoming stimulus or in the timing of an upcoming action. In addition to the range of hundreds of milliseconds, such activity has been recorded in several cortical and subcortical regions in the range of a few seconds, including primary motor and premotor [105] and prefrontal [106], [107] cortices, as well as the thalamus [104]. Some of these data follow a very similar trajectory to those in the hundreds of milliseconds range and a clear distinction between the neural mechanisms coding for similar ranges is not expected [12]. Under our parameters, mean estimates are limited to approximately 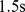, but alternative parameters may furnish estimates of several seconds, potentially unifying some of these data in terms of their underlying mechanism.

Descending neural activity is arguably just as common as climbing activity and is generally regarded as ‘retrospective’ coding (see [7], [73], [108]), *i.e.* neural activity encoding the time since some previous occurrence. In the context of interval timing, the relevant interval can be estimated when the descending activity reaches baseline from an elevated firing rate, initiated by a stimulus (see [32], [96]). Such activity has been recorded in many of the same cortical regions as climbing activity, often in the same experiments (*e.g.* [73], [88], [101], [107]). Other neural data suggest alternative mechanisms for temporal coding in the hundreds of milliseconds range, for example, [109] recorded phasic neural activity in prefrontal cortex, where the firing rate correlated with the length of a preceding interval. In the range of a few seconds, [110] showed that interval recognition and production were predominantly mediated by different PFC neurons, where the latter showed an increase and subsequent decrease in firing rate before subjects indicated their temporal estimates.

### Comparison with earlier neural models of interval timing

A number of neural models have offered mechanistic explanations for temporal coding in recent years. These models can be distinguished along several dimensions, including the temporal range for which they code, the neural and behavioural data for which they provide a mechanistic explanation, the characterization of their dynamics, and whether they function by intracellular or network mechanisms. Most models addressing temporal coding in the range of hundreds of milliseconds to a few seconds are grounded in ascending (climbing) or descending (decaying) neural activity. Several such models have encoded time by neural decay [92], [96], *i.e.* if a stimulus elicits a neural response, then the time after stimulus offset is implicit in the level of activity remaining. Regenerative mechanisms such as recurrent synaptic processing yield network time constants much longer than the time constants of contributing biophysical processes, such as those of membranes and synapses [11], [30], [31], so these temporal codes are not limited to the tens of milliseconds range. Stronger (weaker) intrinsic synapses thus support longer (shorter) temporal estimates, which can be learned by synaptic plasticity [96].

With sufficiently strong recurrent processing, neural activity becomes stable after stimulus offset, *i.e.* attractor states are supported and timing by neural decay is no longer possible. Just outside the attractor regime, however, intervals can be estimated by the time of collapse of quasi-stable activity [93], including compliance with the scalar property [111]. Like our model, this approach makes use of quasi-bistable dynamics, where time is coded by the time of transition from one state to another. The obvious difference between these collapsing-activity models and our model is the direction of state change, but another important difference is the rate of state transitions by individual neurons, described below.

Neural models have simulated ascending activity by a variety of mechanisms, most of which involve attractor dynamics in one form or another. For example, attractor dynamics enable a stimulus-selective population to store the representation of a start cue after its offset, and several models have used such stable, persistent activity as a source of input for the production of climbing activity in a downstream excitatory population. Different models have demonstrated different mechanisms to produce the climbing activity, including slow integration by recurrent synaptic processing [112], short term facilitation at feedforward and recurrent synapes onto excitatory neurons [113], and short term depression at feedforward synapses onto inhibitory interneurons that project to the downstream excitatory population, providing gradual disinhibition [94]. The idea that time can be estimated by integrating regular neural activity has long been deployed in clock-counter models (*a.k.a.* pacemaker-accumulator models), including recent models proposing neural correlates for the required components: an oscillator to provide regular pulses, an integrator to count them, a store to hold a sample interval in memory for comparison with an evolving estimate, and a gate for starting and stopping the timing process (see [114] for extensive review). These models have commonly addressed intervals in the seconds to minutes range and we do not further discuss them here. Suffice to say, persistent mnemonic activity plays a comparable role to the oscillator in models that estimate intervals by the integration of this input to produce climbing activity. The level of background input has been used to similar effect, where stronger (weaker) input produces climbing activity with a steeper (shallower) slope, reaching the threshold sooner (later) and thus estimating shorter (longer) intervals [57], [115]. This mechanism pre-supposes an additional upstream time-sensitive mechanism to govern the strength of input, but differential rates of persistent mnemonic activity in parametric working memory tasks [107] and task-dependent modulation of background cortical spike rates [116] suggest that such a mechanism is plausible. Alternatively, different rates of climbing activity can be produced with a constant mean input if the input variance is integrated [117] or by modulation of recurrent network dynamics, as is the case here.

Providing persistent inputs to integrators is not the only role played by attractor dynamics in models that estimate intervals by climbing to threshold. Indeed, the integrators often utilize attractor dynamics. These models can be differentiated by their number of stable or quasi-stable states and the transitions between them. In neural models incorporating cellular bistability, individual neurons switch rapidly from a down-state, characterized by low rate spiking activity or membrane potential, to an up-state (high rate or membrane potential) when triggered by sufficiently strong input current. Climbing activity occurs if the probability of switching increases with the number of up-state neurons [57], where excitatory recurrent processing creates an avalanche effect in network models [115]. As such, these models assume that climbing activity does not reflect a true gradient of spike rates, but rather, reflects the average of a population of binary neuronal states. This characterization of climbing activity is different from that of our model, where individual neurons in the bump population traverse a gradient of firing rates while climbing to threshold. We confirmed this gradient with the interspike interval (ISI) analysis in [103], where graded firing rates are revealed by a decreasing mean ISI over time and a positively-skewed, unimodal ISI distribution. For all values of the scale factor 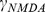, all of these conditions were satisfied in the model (not shown, uni-modality determined by Hartigan's dip test). See [103] for details of the analysis. Our model thus makes the testable prediction that neurons undergoing climbing activity during tasks with a timing requirement will show gradual increases in their firing rates, rather than abrupt transitions.

Like our model, the single-cell model by [56] codes time by climbing to threshold with a true gradient of firing rates. Unlike our model, Durstewitz's model estimates intervals from hundreds of milliseconds to tens of seconds, doing so by an intracellular positive feedback loop between firing rate, voltage-gated calcium influx, and calcium-dependent inward depolarizing current. As described above, fine tuning of this feedback permits firing rate stability along a continuum of rates. Climbing activity is produced when positive feedback provides slightly more current than is required for stability, continually tipping the balance toward higher rates. The amount by which feedback current exceeds the stabilizing current determines the slope of the climbing activity, learned by plasticity at recurrent synapses in the model.

The models by Buonomano and colleagues [95], [99] are based on the premise that the encoding of time in the hundreds of milliseconds range is an inherent property of local cortical circuits, much like our model, but the mechanism by which they do so is very different. Their models do not produce ascending or descending activity, but rather, time is coded implicitly by network state. The long time constants of short term synaptic facilitation and depression in particular (up to 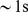) allow the network to reflect its recent history by its current state, which can be readout downstream. This approach provides an elegant solution to the challenge of recognizing rapidly-changing temporal patterns, though it is not clear whether this mechanism could be used for production of temporal estimates. Conversely, our model readily estimates intervals, but is presumably ill-suited to rapid temporal pattern recognition, required, for example, for understanding speech and appreciating music. The synfire chain [118], [119] models by [93], [120] offer another generic-network approach, consistent with a distributed temporal coding framework. In these models, time is coded by the duration of activity propagating through the network, where output cells learn to fire at expected times by synaptic plasticity. Such a timing mechanism could conceivably recognize and produce interval estimates. The above neural models are considered along these and other dimensions in Table 2. See the table caption for further explanation. As a final point on generic circuitry, it seems reasonable to expect a generic mechanism to support multiple functions, such as our demonstration of timing and decision tasks, but we do not expect a single mechanism to execute all possible tasks. While our biophysical network model can be described as a basis function network [28], it is not a universal function approximator [121], [122].

**Table 2:** A comparison of neural models of interval timing.

Model	IL	RA	MS	TG	SP	L
Reutimann et al. (2001)	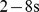	up	net	yes	no	no
Durstewitz (2003)	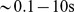	up	cel	yes	no	yes
Miller et al. (2003)	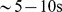	both	net	yes	no	no
Kitano et al. (2003)						
Model 1 (rec. net.)	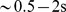	down	both	no	yes	no
Model 2 (synf. ch.)	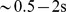	no	no	no	no	yes
Reutimann et al. (2004)	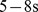	up	net	yes	no	yes
Karmarkar and Buonomano (2007)	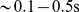	no	no	no	no	yes
Okamoto et al. (2007)	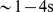	both	both	no	no	no
Okamoto and Hass et al (2008)	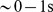	no	no	no	yes	yes
Okamoto and Fukai (2009)	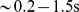	up	both	no	yes	yes
Gavornik et al. (2009)	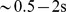	down	no	yes	no	yes
Almeida and Ledberg (2010)						
Model 2 (single cell)	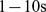	up	cel	no	yes	yes
Model 3 (rec. net.)	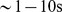	up	net	no	yes	no
Our model	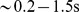	up	net	yes	yes	yes

A comparison of neural models of interval timing. In relation to our model, these models can be distinguished along dimensions including the interval length addressed (IL); whether they show ramping activity, and if so, whether the ramp is ascending or descending (RA: up/down/both/no); whether they rely on multistable dynamics, and if so, whether the mechanism is cellular or network-based (MS: cel/net/both/no); whether neural activity shows a true gradient or an average of binary states (TG: yes/no); whether the scalar property of interval timing was shown: (SP: yes/no); and whether the learning of intervals was shown (L: yes/no).

A number of earlier models have demonstrated the scalar property under different assumptions and mechanisms than our model. In the accumulator network by [123], noisy firing by linear spiking neurons is precisely balanced by random fan-out connectivity, where the scalar property emerges from the Gamma distribution of spiking activity. [124] investigated a multi-cascade structure with multiple memoryless states, *i.e.* a Markov Chain, proving that the system achieves optimal reliability if each state is sequentially and irreversibly activated and generates equal information. In the model by [125], population climbing activity was modelled by an opponent Poisson process, where the scalar property results from the inverse Gaussian distribution of first passage times. The scalar property was also derived in terms of the first passage problem by [111], where in a fully recurrent network of bistable units, the probability of transition from the ‘on’ state to the ‘off’ state increases until network activity collapses. In the models by [57], the scalar property results from the exponential distribution of the transition from the ‘off’ state to the ‘on’ state of bistable units, where the probability of transition is inversely proportional to the duration of the interval being estimated. An information-theoretic framework for classifying timing by a stochastic process is provided by [126], where timing mechanisms that are based on the mean, variance and correlation of the process predict different characteristics of timing errors.

In our model, the interval estimation process is the evolution of network activity to the threshold from an initial state close to the unstable flat steady state. Stronger NMDAR conductance leads to a smaller difference between the initial state and the threshold (see Table 1) and to a larger positive eigenvalue of the unstable flat steady state, producing shorter interval estimates. During the evolution from the initial state to the threshold, noise causes the system to fluctuate around a mean trajectory, where higher levels of noise cause a greater deviation. If the level of noise is independent of the synaptic conductance strength, the deviation will be greater with longer evolution times, so longer estimates will be more variable. Our analysis in Section *The scalar property of interval timing* reveals Weber's law as a necessary property of any timing mechanism that can be expressed as a 1-dimensional OU process with positive drift coefficient (Equation 20), given three constraints: (1) the timing threshold is reduced with increasing feedback excitation, (2) feedback excitation is linear, and (3) the noise in the system is independent of feedback excitation. The first constraint follows from the dominance of NMDARs in local-circuit processing, ie. the state variable of the reduced system is NMDAR activation, which varies much less with increasing NMDAR conductance than the resulting firing rates. Therefore, with increasing conductance, reducing the timing threshold for NMDAR activation preserves the fixed firing threshold in the timing network. The second constraint is common to many analytic reductions of neural models (e.g. [127], [128]). The third constraint can be justified by the long time constant of decay of NMDARs, which allows NMDAR activation to filter synaptic noise at intrinsic synapses, *i.e.* noisy synaptic release is overcome by residual activation.

### Dopamine as a potential mechanism for scaling NMDARs in cortical timing circuitry

The timing network estimates different intervals by the scaling of NMDAR conductance strength by 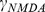, controlling the network's dynamics. Because the strength of cortical NMDAR currents are modulated by dopamine (DA) [59], it is possible that 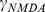 could be instantiated by DA. Further evidence in support of this possibility comes from studies of working memory and interval timing. Working memory, or the active retention of information for use in cognitive tasks, is correlated with persistent mnemonic activity in a number of cortical regions and experimental conditions ([129], [130]). It is widely believed that persistent mnemonic activity is supported by recurrent synaptic processing, where the long time constant of NMDARs is hypothesized to provide an excitatory plateau [131], [132] and to limit network oscillations [133], [134]. This hypothesis is supported by studies showing that NMDAR antagonists abolish cortical up-states in vitro and impair working memory performance [135]. DA antagonists also impair working memory performance and DA is elevated in PFC during working memory tasks [60], [136]. This convergence of evidence has lead to the hypothesis that NMDAR modulation by DA is a crucial factor in controlling attractor dynamics in the service of working memory (see [135]). Our model makes use of the same computational principles, so the possible relationship between 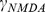 and DA is a compelling one, further supported by the common occurrence of climbing activity during delay periods of working memory tasks (see Discussion section *Prospective and retrospective coding*).

DA is also extensively correlated with interval timing in the seconds to minutes range [13], [137]. In this regard, DA agonists and antagonists are correlated with underestimates and overestimates of intervals respectively (see [114]). Similarly, high and low values of 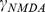 produced short and long interval estimates respectively in our model. Our results therefore suggest that in cortical timing circuitry, DA may strengthen attractor dynamics sufficiently to destablize background states. If so, the slope of climbing activity could be modulated by tonic DA, possibly by the increasing occupancy of D1 receptors due to slow extrasynaptic uptake [138], [139], consistent with enhancement of NMDAR currents via D1 activation [59]. Further work is required to address this possibility. For instance, in addition to modulating NMDAR currents, DA modulates cortical GABAR currents [60], so a more detailed model of DA modulation is required. Furthermore, in the majority of experiments revealing DA involvement in timing in the seconds to minutes range, the effect of DA has been via D2 receptors (see [13], [114]), which act in a largely antagonistic way to D1 receptors [60].

Overall, it is unclear whether DA agonists (antagonists) should be expected to speed up (slow down) the representation of time in the hundreds of milliseconds range, but there are notable gaps in the literature that reveal important lines of enquiry. Firstly, the role of D1 and D2 receptors in timing in the seconds to minutes range appears to be task dependent, as a number of recent studies have shown evidence for D1 involvement in timing in this range, using tasks that were not used in earlier studies showing D2 involvement (*e.g.* [140]–[143]). Additionally, several studies showing D2 involvement in earlier tasks also showed D1 involvement (*e.g.* [144], [145]). Secondly, despite the large body of work addressing D1 and D2 involvement in seconds-to-minutes timing in non-human animals, we are unaware of any such studies investigating the hundreds of milliseconds range (see [146]). Thirdly, despite a growing body of work addressing D2 involvement in timing in healthy humans (see below for studies with clinical populations), we are unaware of any studies to address D1 involvement.

Studies have also shown timing deficits in the seconds to minutes range among patients with Parkinson's Disease ([13], [100]), a pathology characterized by a deterioration of dopaminergic activity [147]. Fewer such studies have considered timing in the hundreds of milliseconds range and results have been mixed among those that have [148]–[150]. Finally, we note that studies addressing the role of DA in interval timing have focused on its effect in the striatum (see [100]). Suffice to say, in addition to their striatal projections, dopaminergic neurons in the basal ganglia project extensively and diffusely to cortex ([151]), so these timing hypotheses are by no means incompatible with our model of local-circuit cortical timing.

### The SAT and an active role for time in decision processing

There has long been an appreciation of the role of time in decision making, where it has been viewed as a medium for filtering noise (see [6] for a historical and mathematical treatment). In sequential sampling models, evidence for each option of a decision is integrated until the accumulated evidence for one of the options reaches a threshold level, at which time the decision is made in favour of that option (see [2]). Because the evidence may be ambiguous and neural processing is noisy, temporal integration provides an average of the evidence, so decisions are not made on the basis of momentary fluctuations. The more time spent integrating, the better the average and the greater the probability of an accurate decision (see [4]). Clearly, speed and accuracy impose conflicting demands within this framework, the reconciliation of which defines the SAT.

In two-choice decision tasks, integrating the difference between the evidence for each option (the decision variable) implements a class of algorithm known as the drift diffusion model (DDM), known to yield the fastest decisions for a given level of accuracy and the most accurate decisions for a given decision time [6]. The DDM thus optimizes speed and accuracy with respect to one another. This approach accounts for a huge volume of experimental data from decision making experiments (see [3]) and under reasonable biophysical constraints, is formally equivalent to models in which neural populations selective for each of two decision options compete via mutual inhibition [128]. Intrinsic synapses support temporal integration in these models [30], [31], [127].

The SAT can be be achieved within this framework by raising and lowering the decision threshold, an approach that readily accounts for behavioural data from decision making tasks (see [5], [6]), but conflicts with neural data showing decision-correlated neural activity that is approximately constant at the time of a decision [9], [97], [152]. A similar mechanism that is potentially consistent with these data is the adjustment of the initial level of neural activity on which the decision variable builds, a possibility that is supported by recent functional magnetic resonance imaging (fMRI) studies [153], [154]. Such a mechanism requires a means to control the baseline level of activation in decision circuitry, but this requirement could be satisfied by spatially non-selective input, potentially instantiated by the persistent encoding of task requirements in PFC (see [155]). Neural models have demonstrated the SAT under this approach [24], [128]. Another potential means of trading speed and accuracy with a fixed neural threshold is the adjustment of the strength of synapses onto downstream neurons reading out or implementing the decision [156]. It is not clear, however, that the timescale of plasticity processes is consistent with the rapidity with which experimental subjects trade speed and accuracy from trial to trial [24].

An alternative, compatible mechanism is that decision-makers explicitly encode their temporal constraints, controlling the SAT downstream (Figures 10 and 11). Several recent studies have considered such an active role for the representation of time in decision making. For example, the DDM has been augmented with time-dependent mechanisms [8], [10]. The fundamental difference between these and earlier diffusion models is that the representation of elapsed time has an increasing influence on decision processing as each trial progresses, sometimes referred to as an ‘urgency’ signal. In the time-variant DDM by [157], amplifying the input by a growing temporal signal was shown to earn more reward per unit time than the standard DDM. This approach is functionally equivalent to lowering the decision threshold over the course of each trial, where later evidence is more heavily weighted than earlier evidence at the expense of a decreasing signal-to-noise ratio [8]. Conversely, if the incoming evidence and the evolving decision variable are both amplified by the temporal signal, there is a transition from a heavier weighting of the former to the latter [11], similar to the transition from extrinsic to intrinsic processing hypothesized to underlie local-circuit cortical processing (see [29]). We hypothesize that climbing activity drives the rate of this transition in downstream decision circuitry, controlling the SAT. This hypothesis is consistent with neural data from experimental tasks with a timing requirement on the relevant order for perceptual decisions (see the previous section) and with neural and behavioural data revealing the SAT (see [5]). It is also consistent with neural data showing a fixed decision threshold [9], [97], [152]. Indeed, at least one experiment has reported climbing activity that was correlated with the time of decisions in a perceptual task, but not with the evidence [9]. Such activity effectively encodes elapsed time relative to an estimated interval [32], shown recently to earn more reward per unit time than a persistent, top-down signal in a more abstract network model than the one used here, where the temporal signal was a linear function of time [11].

#### A transition through dynamic regimes during decisions

We have previously described the dynamics of the decision network within this framework, where a linear ramping signal was shown to drive a transition from a leakage-dominated regime to an inhibition-dominated regime in a simplified version of the decision network used here [11]. In the leakage regime, the network quickly reaches a steady state where the decision variable leaks away at the same rate as it accumulates, whereas in the inhibition regime, the decision variable is amplified by strong recurrent dynamics, rapidly driving the network toward an attractor corresponding to the target or the distractor, *i.e.* activity in one population inhibits activity in the other. In either extreme case, the effective time constant of the network is short and therefore unsuitable for temporal integration. However, gain modulation by the evolving timing signal gradually stretches the time constant prior to the bifurcation between regimes, facilitating a progression through processing stages that correspond to noise filtering, integration of evidence, amplification of integrated evidence, and choice selection. Effectively, the network implements late amplification of a high quality decision variable, accrued by early conservatism. The rate at which the timing signal ramps up determines the rate of the progression. Since the network always goes through the same stages, decision-selective firing rates are fixed at decision time, consistent with neural data [9], [97], [152]. The SAT is thus achieved by estimating elapsed time relative to a deadline. Under accuracy (speed) conditions, the decision network spends more (less) time in regimes with a longer (shorter) time constant, integrating more (less) evidence at the expense of speed (accuracy). Here, the timing network provides the temporal signal by virtue of its unstable background state, implementing gain modulation by spatially non-selective, additive input [33] (Figure 1C). This time-dependent approach leads to more accurate decisions per unit time than the modulation of decision dynamics by a time-independent signal because at the time of the bifurcation, the state of the decision network is closer to the attractor corresponding to the target than to the attractor corresponding to the distractor. We confirmed this analysis for the present, biophysically motivated model by running decision trials without the timing network for a range of values of 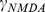 in the decision network, where these values spanned slow and fast decision times. Without the timing network, the decision network systematically earned less reward than the coupled-circuit model, according to the reward rate definitions of [158] and [6] (not shown).

#### The weighting of evidence during decisions

The dynamics of the decision network under time-variant gain modulation lead to a testable prediction about the weighting of evidence at different times during decisions. This prediction differs from those of earlier time-*invariant* approaches to the SAT [5], [24]. As described above (see *The SAT and an active role for time in decision processing*), neural models in which stimulus-selective populations compete via mutual inhibition can be approximated by the DDM [128]. These models (including ours) are based on the premise that intrinsic (recurrent) synapses underlie the buildup of evidence [30], so the decision variable accumulates not only by the sequential sampling of instantaneous evidence, but also by a factor of its present value at each instant in time. These models are thus equivalent to a 1-dimensional OU diffusion process
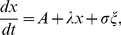
(28)where 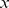 is the decision variable, 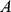 is the drift (here the mean difference between the target and distractor signals), 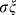 is white noise with standard deviation 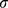, and 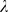 scales the present value of the decision variable. The difference between our model and earlier neural models of the SAT can be described in terms of 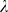. As described in [128], 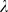 equals 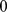 in the standard DDM, so Equation 28 reduces to a Wiener diffusion process. With negative 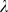, the model is dominated by leakage and converges to a stable fix point with mean 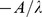. With positive 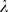, the fix point is unstable and 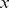 is repelled from it at a rate that depends on 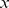 (faster for larger 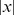). The neural model in [24] trades speed and accuracy by the modulation of network dynamics by a constant, spatially non-selective signal during each trial, *i.e.* the signal ‘locks in’ the dynamics for a given trial, equivalent to adjusting 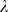 between trials in Equation 28. Under this approach, evidence is equally weighted over the full trial for 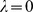. Negative 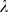 produces a recency effect, where later evidence has greater weight than earlier evidence because the latter has more time to leak away. Positive 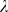 produces a primacy effect, where earlier evidence has more weight than later evidence because the former is amplified for longer. See [127] for further discussion of these principles. In a task in which the mean evidence changes systematically within each trial (e.g. [10], [159]), time-invariant models can either predict a recency effect, a primacy effect, or uniform weighting of evidence.

Expressing our model according to Equation 28 requires that 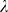 be time-dependent, so our model becomes 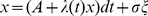, where 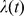 increases monotonically from a negative value to a positive value as the temporal estimate evolves within each trial. Under this approach, evidence is most heavily weighted during the transition from negative to positive 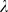, *i.e.* in the middle of the trial [11]. These different predictions could be tested by briefly changing the mean evidence favouring the target and distractor at different times during a decision trial.

### Summary, conclusions and future work

Despite the longstanding attribution of a prominent role for time in perceptual decisions and the growing appreciation of the role of temporal codes in behaviour more generally, few studies have considered the interactions between spatial and temporal codes in decision making. The SAT provides a potential window into these interactions, but most theories of the SAT have ignored the encoding of time, with other factors limiting the amount of time spent integrating evidence (see [5]). We hypothesize a compatible mechanism: the SAT can be accomplished by estimating one's temporal constraints (Figures 10 and 11), where climbing activity encodes these estimates (Figures 2 and 6) and controls the SAT by gain modulation (Figures 10 and 11). This hypothesis is consistent with a growing body of neural data from tasks with a timing requirement (see above) and with the notion of urgency in decision tasks [9], [10]. Our implementation of the same network for timing and decision making is consistent with the network's foundations as a generic, local-circuit cortical model [27]–[29] and with a framework of distributed, generic cortical timing circuitry [12]. In this regard, we have demonstrated a plausible framework for spatiotemporal integration in cortex: the modulation of spatially selective, temporally non-selective processing by temporally selective, spatially non-selective processing. While the present implementation of this framework is uni-directional, the mutual (bidirectional) influence of spatial and temporal codes is an interesting future direction.

The variability of decision times in perceptual tasks has been an important means of characterizing decision processing (see [2], [77]). Our study suggests that the encoding of temporal constraints is an important source of this variability. Despite the large body of work characterizing the variability of temporal estimates on the relevant order and the broad range of variability across these different experimental paradigms (see [55]), we are unaware of any studies that have systematically controlled timing variability in a given task, except by varying the length of the intervals being estimated. Our study suggests that such an approach would not only help to characterize the mechanisms underlying temporal coding, but would further characterize decision making and its relationship with time. In recent years, there has been growing interest in the optimality of decisions in terms of reward-maximization [6], [156]–[158] and a compelling possibility is that decision makers maximize reward rate by estimating their deadlines [11]. It is conceivable that estimates of deadlines imposed by the environment will vary differently than self-imposed deadlines on the same temporal order, a possibility that could be addressed by controlling the time available to respond and the timing of reward in decision tasks. Future work should address this possibility.

## References

[1] Schall JD (2001) Neural basis of deciding, choosing and acting. Nature Reviews Neuroscience 2: 33–42. 11253357 10.1038/35049054

[2] Smith PL, Ratcliff R (2004) Psychology and neurobiology of simple decisions. Trends in Neurosciences 27: 161–168. 15036882 10.1016/j.tins.2004.01.006

[3] Gold JI, Shadlen MN (2007) The neural basis of decision making. Annual Review of Neuroscience 30: 535–574. 10.1146/annurev.neuro.29.051605.113038 17600525

[4] Bogacz R (2007) Optimal decision-making theories: linking neurobiology with behaviour. Trends in Cognitive Sciences 11: 118–125. 17276130 10.1016/j.tics.2006.12.006

[5] Bogacz R, Wagenmakers EJ, Forstmann BU, Nieuwenhuis S (2010) The neural basis of the speed accuracy tradeoff. Trends in Neurosciences 33: 10–16. 19819033 10.1016/j.tins.2009.09.002

[6] Gold JI, Shadlen MN (2002) Banburismus and the brain: Decoding the relationship between sensory stimuli, decisions, and reward. Neuron 36: 299–308. 12383783 10.1016/s0896-6273(02)00971-6

[7] Durstewitz D, Deco G (2008) Computational significance of transient dynamics in cortical networks. European Journal of Neuroscience 27: 217–227. 18093174 10.1111/j.1460-9568.2007.05976.x

[8] Ditterich J (2006) Stochastic models of decisions about motion direction: Behavior and physiology. Neural Networks 19: 981–1012. 16952441 10.1016/j.neunet.2006.05.042

[9] Churchland AK, Kiani R, Shadlen MN (2008) Decision-making with multiple alternatives. Nature Neuroscience 11: 693–702. 18488024 10.1038/nn.2123 PMC2453226

[10] Cisek P, Puskas GA, El-Murr S (2009) Decisions in changing conditions: The urgency-gating model. The Journal of Neuroscience 29: 11560–11571. 19759303 10.1523/JNEUROSCI.1844-09.2009 PMC6665752

[11] Standage D, You H, Wang DH, Dorris MC (2011) Gain modulation by an urgency signal controls the speed-accuracy trade-off in a network model of a cortical decision circuit. Frontiers in Computational Neuroscience 5: 1–14. 21415911 10.3389/fncom.2011.00007 PMC3042674

[12] Mauk MD, Buonomano DV (2004) The neural basis of temporal processing. Annual Review of Neuroscience 27: 307–340. 10.1146/annurev.neuro.27.070203.144247 15217335

[13] Buhusi CV, Meck WH (2005) What makes us tick? Functional and neural mechanisms of interval timing. Nature Reviews Neuroscience 6: 755–765. 16163383 10.1038/nrn1764

[14] Glimcher PW (2003) The neurobiology of visual-saccadic decision making. Annual Review of Neuroscience 26: 133–179. 10.1146/annurev.neuro.26.010302.081134 14527268

[15] Buonomano DV, Karmarkar UR (2002) How do we tell time? The Neuroscientist 8: 42–51. 11843098 10.1177/107385840200800109

[16] Wittmann M (2009) The inner experience of time. Philosophical Transactions of the Royal Society 364: 1955–1967. 10.1098/rstb.2009.0003 PMC2685813 19487197

[17] Ivry RB, Spencer RMC (2004) The neural representation of time. Current Opinion in Neurobiology 14: 225–232. 15082329 10.1016/j.conb.2004.03.013

[18] Buonomano DV, Bramen J, Khodadadifar M (2009) Influence of the interstimulus interval on temporal processing and learning: testing the state-dependent network model. Philosophical transactions of the Royal Society 364: 1865–1873. 10.1098/rstb.2009.0019 PMC2685819 19487189

[19] Douglas RJ, Martin KAC (2004) Neuronal circuits of the neocortex. Annual Review of Neuro- science 27: 419–451. 10.1146/annurev.neuro.27.070203.144152 15217339

[20] Somers DC, Nelson SB, Sur M (1995) An emergent model of orientation selectivity in cat visual cortical simple cells. The Journal of Neuroscience 15: 5448–5465. 7643194 10.1523/JNEUROSCI.15-08-05448.1995 PMC6577625

[21] Compte A, Brunel N, Goldman-Rakic PS, Wang XJ (2000) Synaptic mechanisms and network dynamics underlying spatial working memory in a cortical network model. Cerebral Cortex 10: 910–923. 10982751 10.1093/cercor/10.9.910

[22] Gutkin BS, Laing CR, Colby CL, Chow CC, Ermentrout BG (2001) Turning on and off with excitation: The role of spike-timing asynchrony and synchrony in sustained neural activity. Journal of Computational Neuroscience 11: 121–134. 11717529 10.1023/a:1012837415096

[23] Ma WJ, Beck JM, Latham PE, Pouget A (2006) Bayesian inference with probabilistic population codes. Nature Neuroscience 9: 1432–1438. 17057707 10.1038/nn1790

[24] Furman M, Wang XJ (2008) Similarity effect and optimal control of multiple-choice decision making. Neuron 60: 1153–1168. 19109918 10.1016/j.neuron.2008.12.003 PMC2633638

[25] York LC, van Rossum MCW (2009) Recurrent networks with short term synaptic depression. Journal of Computational Neuroscience 27: 607–620. 19578989 10.1007/s10827-009-0172-4

[26] Standage D, Pare M (2011) Persistent storage capability impairs decision making in a biophysical network model. Neural Networks 24: 1062–1073. 21658905 10.1016/j.neunet.2011.05.004

[27] Wilson HR, Cowan JD (1973) A mathematical theory of the functional dynamics of cortical and thalamic nervous tissue. Kybernetik 13: 55–80. 4767470 10.1007/BF00288786

[28] Pouget A, Dayan P, Zemel R (2000) Information processing with population codes. Nature Reviews Neuroscience 1: 125–132. 11252775 10.1038/35039062

[29] Douglas RJ, Martin KAC (2007) Recurrent neuronal circuits in the neocortex. Current Biology 17: R496–R500. 17610826 10.1016/j.cub.2007.04.024

[30] Wang XJ (2002) Probabilistic decision making by slow reverberation in cortical circuits. Neuron 36: 955–968. 12467598 10.1016/s0896-6273(02)01092-9

[31] Wong KF, Wang XJ (2006) A recurrent network mechanism of time integration in perceptual decisions. The Journal of Neuroscience 26: 1314–1328. 16436619 10.1523/JNEUROSCI.3733-05.2006 PMC6674568

[32] Durstewitz D (2004) Neural representation of interval time. NeuroReport 15: 745–749. 15073507 10.1097/00001756-200404090-00001

[33] Salinas E, Abbott LF (1996) A model of multiplicative neural responses in parietal cortex. Proceedings of the National Academy of Sciences of the United States of America 93: 11956–11961. 8876244 10.1073/pnas.93.21.11956 PMC38165

[34] Salinas E, Thier P (2000) Gain modulation: A major computational principle of the central nervous system. Neuron 27: 15–21. 10939327 10.1016/s0896-6273(00)00004-0

[35] Salinas E, Sejnowski TJ (2001) Gain modulation in the central nervous system: Where behavior, neurophysiology, and computation meet. The Neuroscientist 7: 430–440. 11597102 10.1177/107385840100700512 PMC2887717

[36] Tuckwell H (1988) Introduction to theoretical neurobiology. Cambridge: Cambridge University Press.

[37] Hellwig B (2000) A quantitative analysis of the local connectivity between pyramidal neurons in layers 2/3 of the rat visual cortex. Biological Cybernetics 82: 111–121. 10664098 10.1007/PL00007964

[38] Voges N, Schuz A, Aertsen A, Rotter S (2010) A modelers view on the spatial structure of intrinsic horizontal connectivity in the neocortex. Progress in Neurobiology 92: 277–292. 20685378 10.1016/j.pneurobio.2010.05.001

[39] Mountcastle VB (1997) The columnar organization of the neocortex. Brain 120: 701–722. 9153131 10.1093/brain/120.4.701

[40] Sompolinsky H, Shapley R (1997) New perspectives on the mechanisms for orientation selectivity. Current Opinion in Neurobiology 7: 514–522. 9287203 10.1016/s0959-4388(97)80031-1

[41] Jahr CE, Stevens CF (1990) Voltage dependence of nmda-activated macroscopic conductances predicted by single-channel kinetics. The Journal of Neuroscience 10: 3178–3182. 1697902 10.1523/JNEUROSCI.10-09-03178.1990 PMC6570236

[42] Maria Cecilia Angulo JR, Audinat E (1999) Postsynaptic glutamate receptors and integrative properties of fast-spiking interneurons in the rat neocortex. The Journal of Neuroscience 82: 1295–1302. 10.1152/jn.1999.82.3.1295 10482748

[43] Desai NS, Cudmore RH, Nelson SB, Turrigiano GG (2002) Critical periods for experience-dependent synaptic scaling in visual cortex. Nature Neuroscience 5: 783–789. 12080341 10.1038/nn878

[44] Hestrin S (1993) Different glutamate receptor channels mediate fast excitatory synaptic currents in inhibitory and excitatory cortical neurons. Neuron 11: 1083–1091. 7506044 10.1016/0896-6273(93)90221-c

[45] JMcBain C, Fisahn A (2001) Interneurons unbound. Nature Reviews Neuroscience 2: 11–23. 11253355 10.1038/35049047

[46] Hull C, Isaacson JS, Scanziani M (2009) Postsynaptic mechanisms govern the differential excitation of cortical neurons by thalamic inputs. The Journal of Neuroscience 29: 9127–9136. 19605650 10.1523/JNEUROSCI.5971-08.2009 PMC2753516

[47] Berretta N, Jones RSG (1996) A comparison of spontaneous epscs in layer ii and layer iv-v neurons of the rat entorhinal cortex in vitro. Journal of Neurophysiology 76: 1089–1100. 8871222 10.1152/jn.1996.76.2.1089

[48] Povysheva NV, Gonzalez-Burgos G, Zaitsev AV, Kroner S, Barrionuevo G, et al (2006) Properties of excitatory synaptic responses in fast-spiking interneurons and pyramidal cells from monkey and rat prefrontal cortex. Cerebral Cortex 16: 541–552. 16033926 10.1093/cercor/bhj002

[49] Salin PA, Prince DA (1996) Spontaneous gaba a receptor-mediated inhibitory currents in adult rat somatosensory cortex. Journal of Neurophysiology 75: 1573–1588. 8727397 10.1152/jn.1996.75.4.1573

[50] Xiang Z, Huguenard JR, Prince DA (1998) Gaba a receptor-mediated currents in interneurons and pyramidal cells of rat visual cortex. Journal of Physiology 506: 715–730. 9503333 10.1111/j.1469-7793.1998.715bv.x PMC2230760

[51] Markram H, Toledo-Rodriguez M, Wang Y, Gupta A, Gilad, et al (2004) Interneurons of the neocortical inhibitory system. Nature Reviews Neuroscience 5: 793–807. 15378039 10.1038/nrn1519

[52] Destexhe A, Rudolph M, Fellous JM, Sejnowski T (2001) Fluctuating synaptic conductances recreate in vivo-like activity in neocortical neurons. Neuroscience 107: 13–24. 11744242 10.1016/s0306-4522(01)00344-x PMC3320220

[53] Thompson K, Hanes D, Bichot N, Schall J (1996) Perceptual and motor processing stages identified in the activity of macaque frontal eye field. Journal of Neurophysiology 76: 440–455. 10.1152/jn.1996.76.6.4040 8985899

[54] Wilson HR, Cowan JD (1972) Excitatory and inhibitory interactions in localized populations of model neurons. Biophysical Journal 12: 1–24. 4332108 10.1016/S0006-3495(72)86068-5 PMC1484078

[55] Gibbon J, Malapani C, Dale CL, Gallistel CR (1997) Toward a neurobiology of temporal cognition: advances and challenges. Current Opinion in Neurobiology 7: 170–184. 9142762 10.1016/s0959-4388(97)80005-0

[56] Durstewitz D (2003) Self-organizing neural integrator predicts interval times through climbing activity. The Journal of Neuroscience 23: 5342–5353. 12832560 10.1523/JNEUROSCI.23-12-05342.2003 PMC6741165

[57] Almeida R, Ledberg A (2010) A biologically plausible model of time-scale invariant interval timing. Journal of Computational Neuroscience 28: 155–175. 19862610 10.1007/s10827-009-0197-8 PMC2825317

[58] Destexhe A, Pare D (1999) Impact of network activity on the integrative properties of neocortical pyramidal neurons in vivo. Journal of Neurophysiology 81: 1531–1547. 10200189 10.1152/jn.1999.81.4.1531

[59] Seamans JK, Durstewitz D, Christie BR, Stevens CF, Sejnowski TJ (2001) Dopamine d1/d5 receptor modulation of excitatory synaptic inputs to layer v prefrontal cortex neurons. Proceedings of the National Academy of Sciences of the United States of America 98: 301–306. 11134516 10.1073/pnas.011518798 PMC14585

[60] Seamans JK, Yang CR (2004) The principal features and mechanisms of dopamine modulation in the prefrontal cortex. Progress in Neurobiology 74: 1–57. 15381316 10.1016/j.pneurobio.2004.05.006

[61] Fung CCA, Wong KYM, Wu S (2010) A moving bump in a continuous manifold: A comprehensive study of the tracking dynamics of continuous attractor neural networks. Neural Computation 22: 752–792. 19922292 10.1162/neco.2009.07-08-824

[62] Gibbon J (1977) Scalar expectancy theory and weber's law in animal timing. Psychological Review 84: 279–325.

[63] Wearden JH, Lejeune H (2007) Scalar properties in human timing: Conformity and violations. The Quarterly Journal of Experimental Psychology 0: 1–19. 10.1080/17470210701282576 18938276

[64] Lejeune H, Wearden JH (2006) Scalar properties in animal timing: Conformity and violations. The Quarterly Journal of Experimental Psychology 59: 1875–1908. 16987779 10.1080/17470210600784649

[65] Lewis P, Miall R (2009) The precision of temporal judgement: milliseconds, many minutes, and beyond. Philosophical transactions of the Royal Society 364: 1897–1905. 10.1098/rstb.2009.0020 PMC2685820 19487192

[66] Wearden JH, McShane B (1988) Interval production as an analogue of the peak procedure: Evidence for similarity of human and animal timing processes. The Quarterly Journal of Experimental Psychology 40B: 363–375.

[67] Merchant H, Zarco W, Prado L (2008) Do we have a common mechanism for measuring time in the hundreds of millisecond range? Evidence from multiple-interval timing tasks. Journal of Neurophysiology 99: 939–949. 18094101 10.1152/jn.01225.2007

[68] Strogatz S (2001) Nonlinear dynamics and chaos: with applications to physics, biology, chemistry, and engineering. New York: Perseus Books Group.

[69] Miller EK, Erickson CA, Desimone R (1996) Neural mechanisms of visual working memory in prefrontal cortex of the macaque. Journal of Neuroscience 16: 5154–5167. 8756444 10.1523/JNEUROSCI.16-16-05154.1996 PMC6579322

[70] Callaway EM (2004) Feedforward, feedback and inhibitory connections in primate visual cortex. Neural Networks 17: 625–632. 15288888 10.1016/j.neunet.2004.04.004

[71] PMunoz D, Everling S (2004) Look away: the anti-saccade task and the voluntary control of eye movement. Nature Reviews Neuroscience 5: 218–228. 14976521 10.1038/nrn1345

[72] Lo CC, Boucher L, Pare M, Xiao Jing Wang JDS (2009) Proactive inhibitory control and attractor dynamics in countermanding action: A spiking neural circuit model. The Journal of Neuroscience 29: 9059–9071. 19605643 10.1523/JNEUROSCI.6164-08.2009 PMC2756461

[73] Rainer G, Rao SC, Miller EK (1999) Prospective coding for objects in primate prefrontal cortex. Journal of Neuroscience 19: 5493–5505. 10377358 10.1523/JNEUROSCI.19-13-05493.1999 PMC6782318

[74] Wang H, III GGS, Wang XJ, Gaoa WJ (2008) A specialized nmda receptor function in layer 5 recurrent microcircuitry of the adult rat prefrontal cortex. Proceedings of the National Academy of Sciences of the United States of America 105: 16791–16796. 18922773 10.1073/pnas.0804318105 PMC2575498

[75] Chen G, Greengard P, Yan Z (2003) Potentiation of nmda receptor currents by dopamine d1 receptors in prefrontal cortex. Proceedings of the National Academy of Sciences of the United States of America 101: 2596–2600. 10.1073/pnas.0308618100 PMC356995 14983054

[76] Prescott SA, Koninck YD (2003) Gain control of firing rate by shunting inhibition: Roles of synaptic noise and dendritic saturation. Proceedings of the National Academy of Sciences of the United States of America 100: 2076–2081. 12569169 10.1073/pnas.0337591100 PMC149961

[77] Ratcliff R, Smith PL (2004) A comparison of sequential sampling models for two-choice reaction time. Psychological Review 111: 333–367. 15065913 10.1037/0033-295X.111.2.333 PMC1440925

[78] Wang XJ (2008) Decision making in recurrent neuronal circuits. Neuron 60: 215–234. 18957215 10.1016/j.neuron.2008.09.034 PMC2710297

[79] Ratcliff R, McKoon G (2008) The diffusion decision model: theory and data for two-choice decision tasks. Neural Computation 20: 873–922. 18085991 10.1162/neco.2008.12-06-420 PMC2474742

[80] Ivry RB (1996) The representation of temporal information in perception and motor control. Current Opinion in Neurobiology 6: 851–857. 9000026 10.1016/s0959-4388(96)80037-7

[81] Grondin S (2001) From physical time to the first and second moments of psychological time. Psychological Bulletin 127: 22–44. 11271754 10.1037/0033-2909.127.1.22

[82] Getty DJ (1975) Discrimination of short temporal intervals: a comparison of two models. Perception and Psychophysics 18: 1–8.

[83] Ivry RB, Hazeltine RE (1995) Perception and production of temporal intervals across a range of durations: evidence for a common timing mechanism. Journal of Experimental Psychology 21: 3–18. 10.1037//0096-1523.21.1.3 7707031

[84] Niki H, Watanabe M (1979) Prefrontal and cingulate unit activity during timing behavior in the monkey. Brain Research 171: 213–224. 111772 10.1016/0006-8993(79)90328-7

[85] Mauritz KH, Wise SP (1986) Premotor cortex of the rhesus monkey: neuronal activity in anticipation of predictable environmental events. Experimental Brain Research 61: 229–244. 3948938 10.1007/BF00239513

[86] Constantinidis C, Steinmetz MA (1996) Neuronal activity in posterior parietal area 7a during the delay periods of a spatial memory task. Journal of Neurophysiology 76: 1352–1355. 8871242 10.1152/jn.1996.76.2.1352

[87] Leon MI, Shadlen MN (2003) Representation of time by neurons in the posterior parietal cortex of the macaque. Neuron 38: 317–327. 12718864 10.1016/s0896-6273(03)00185-5

[88] Shuler MG, Bear MF (2006) Reward timing in the primary visual cortex. Science 311: 1606–1609. 16543459 10.1126/science.1123513

[89] Maimon G, Assad JA (2006) A cognitive signal for the proactive timing of action in macaque lip. Nature Neuroscience 9: 948–955. 16751764 10.1038/nn1716

[90] Lewis PA, Miall RC (2003) Distinct systems for automatic and cognitively controlled time measurement: evidence from neuroimaging. Current Opinion in Neurobiology 13: 250–255. 12744981 10.1016/s0959-4388(03)00036-9

[91] Coull JT, Nobre AC (2008) Dissociating explicit timing from temporal expectation with fmri. Current Opinion in Neurobiology 18: 137–144. 18692573 10.1016/j.conb.2008.07.011

[92] Hopfield JJ, Brody CD (2001) What is a moment? Transient synchrony as a collective mechanism for spatiotemporal integration. Proceedings of the National Academy of Sciences of the United States of America 98: 1282–1287. 11158631 10.1073/pnas.031567098 PMC14746

[93] Kitano K, Okamoto H, Fukai T (2003) Time representing cortical activities: two models inspired by prefrontal persistent activity. Biological Cybernetics 88: 387–394. 12750901 10.1007/s00422-002-0390-6

[94] Reutimann J, Yakovlev V, Fusi S, Senn W (2004) Climbing neuronal activity as an event-based cortical representation of time. The Journal of Neuroscience 24: 3295–3303. 15056709 10.1523/JNEUROSCI.4098-03.2004 PMC6730018

[95] Karmarkar UR, Buonomano DV (2007) Timing in the absence of clocks: encoding time in neural network states. Neuron 53: 427–438. 17270738 10.1016/j.neuron.2007.01.006 PMC1857310

[96] Gavornik JP, Shuler MGH, Loewenstein Y, Bear MF, Shouval HZ (2009) Learning reward timing in cortex through reward dependent expression of synaptic plasticity. Proceedings of the National Academy of Sciences of the United States of America 106: 6826–6831. 19346478 10.1073/pnas.0901835106 PMC2672535

[97] Roitman JD, Shadlen MN (2002) Response of neurons in the lateral intraparietal area during a combined visual discrimination reaction time task. The Journal of Neuroscience 22: 9475–9489. 12417672 10.1523/JNEUROSCI.22-21-09475.2002 PMC6758024

[98] Shen K, Kalwarowsky S, Clarence W, Brunamonti E, Pare M (2010) Beneficial effects of the nmda antagonist ketamine on decision processes in visual search. Journal of Neuroscience 30: 9947–9953. 20660277 10.1523/JNEUROSCI.6317-09.2010 PMC6632808

[99] Buonomano DV, Merzenich MM (1995) Temporal information transformed into a spatial code by a neural network with realistic properties. Science 267: 1028–1030. 7863330 10.1126/science.7863330

[100] Meck WH, Penney TB, Pouthas V (2008) Cortico-striatal representation of time in animals and humans. Current Opinion in Neurobiology 18: 145–152. 18708142 10.1016/j.conb.2008.08.002

[101] Isomura Y, Ito Y, Akazawa T, Nambu A, Takada M (2003) Neural coding of “attention for action” and “response selection” in primate anterior cingulate cortex. The Journal of Neuroscience 23: 8002–8012. 12954861 10.1523/JNEUROSCI.23-22-08002.2003 PMC6740492

[102] Janssen P, Shadlen MN (2005) A representation of the hazard rate of elapsed time in macaque area LIP. Nature Neuroscience 8: 234–241. 15657597 10.1038/nn1386

[103] Thevarajah D, Mikulic A, Dorris MC (2009) Role of the superior colliculus in choosing mixed-strategy saccades. Journal of Neuroscience 29: 1998–2008. 19228954 10.1523/JNEUROSCI.4764-08.2009 PMC6666345

[104] Komura Y, Tamura R, Uwano T, Nishijo H, Kaga K, et al (2001) Retrospective and prospective coding for predicted reward in the sensory thalamus. Nature 412: 546–549. 11484055 10.1038/35087595

[105] Lebedev MA, O'Doherty JE, Nicolelis MAL (2008) Decoding of temporal intervals from cortical ensemble activity. Journal of Neurophysiology 99: 166–186. 18003881 10.1152/jn.00734.2007

[106] Kojima S, Goldman-Rakic PS (1982) Delay-related activity of prefrontal neurons in rhesus monkeys performing delayed response. Brain Research 248: 43–49. 7127141 10.1016/0006-8993(82)91145-3

[107] Brody CD, Hernandez A, Zainos A, Romo R (2003) Timing and neural encoding of somatosensory parametric working memory in macaque prefrontal cortex. Cerebral Cortex 13: 1196–1207. 14576211 10.1093/cercor/bhg100

[108] Quintana J, Fuster JM (1992) Mnemonic and predictive functions of cortical neurons in a memory task. Neuroreport 3: 721–724. 1520863 10.1097/00001756-199208000-00018

[109] Genovesio A, Tsujimoto S, Wise SP (2006) Neuronal activity related to elapsed time in prefrontal cortex. Journal of Neurophysiology 95: 3281–3285. 16421197 10.1152/jn.01011.2005 PMC1475947

[110] Yumoto N, Lu X, Henry TR, Miyachi S, Nambu A, et al (2011) A neural correlate of the processing of multi-second time intervals in primate prefrontal cortex. PLoS One 6: 1–7. 10.1371/journal.pone.0019168 PMC3083430 21556372

[111] Okamoto H, Fukai T (2001) Neural mechanism for a cognitive timer. Physical Review Letters 86: 3919–3922. 11329357 10.1103/PhysRevLett.86.3919

[112] Miller P, Brody CD, Romo R, Wang XJ (2003) A recurrent network model of somatosensory parametric working memory in the prefrontal cortex. Cerebral Cortex 13: 1208–1218. 14576212 10.1093/cercor/bhg101 PMC4632206

[113] Reutimann J, Fusi S, Senn W, Yakovlev V, Zohary E (2001) A model of expectation effects in inferior temporal cortex. Neurocomputing 38–40: 1533–1540.

[114] Matell MS, Meck WH (2004) Cortico-striatal circuits and interval timing: coincidence detection of oscillatory processes. Cognitive Brain Research 21: 139–170. 15464348 10.1016/j.cogbrainres.2004.06.012

[115] Okamoto H, Isomura Y, Takada M, Fukai T (2007) Temporal integration by stochastic recurrent network dynamics with bimodal neurons. Journal of Neurophysiology 97: 3859–3867. 17392417 10.1152/jn.01100.2006

[116] Luck SJ, Chelazzi L, Hillyard SA, Desimone R (1997) Neural mechanisms of spatial selective attention in areas v1, v2, and v4 of macaque visual cortex. Journal of Neurophysiology 77: 24–42. 9120566 10.1152/jn.1997.77.1.24

[117] Okamoto H, Fukai T (2009) recurrent network models for perfect temporal integration of fluctuating correlated inputs. Public Library of Science Computational Biology 5: 1–10. 10.1371/journal.pcbi.1000404 PMC2685482 19503816

[118] Abeles M (1991) Corticonics: Neural Circuits of the Cerebral Cortex. Cambridge, UK: Cambridge University Press.

[119] Abeles M, Bergman H, Margalit E, Vaadia E (1993) Spatiotemporal firing patterns in the frontal cortex of behaving monkeys. Journal of Neurophysiology 70: 1629–1638. 8283219 10.1152/jn.1993.70.4.1629

[120] Hass J, Blaschke S, Rammsayer T, Herrmann JM (2008) A neurocomputational model for optimal temporal processing. Journal of Computational Neuroscience 25: 449–464. 18379866 10.1007/s10827-008-0088-4

[121] Cybenko G (1989) Approximation by superpositions of a sigmoidal function. Mathematics of Control, Signals, and Systems 2: 303–314.

[122] Hornik K (1991) Approximation capabilities of multi-layer feedforward networks. Neural Networks 4: 251–257.

[123] Shapiro JL, Wearden J (2002) Reinforcement learning and time perception - a model of animal experiments. In: Ditterich TG, Becker S, Ghahramani Z, editors, Advances in Neural Information Processing Systems (NIPS), Cambridge, MA: MIT Press. pp. 115–122.

[124] Escola S, Fontanini A, Katz D, Paninski L (2009) Hidden markov models for the stimulus-response relationships of multistate neural systems. Neural Computation 23: 1071–1132. 10.1162/NECO_a_00118 21299424

[125] Simen P, Balci F, deSouza L, Cohen JD, Holmes P (2011) A model of interval timing by neural integration. The Journal of Neuroscience 31: 9238–9253. 21697374 10.1523/JNEUROSCI.3121-10.2011 PMC3142662

[126] Hass J, Herrmann JM (2012) The neural representation of time: An information-theoretic perspective. Neural Computation 24: 1519–1552. 22364498 10.1162/NECO_a_00280

[127] Usher M, McClelland JL (2001) On the time course of perceptual choice: The leaky competing accumulator model. Psychological Review 108: 550–592. 11488378 10.1037/0033-295x.108.3.550

[128] Bogacz R, Brown E, Moehlis J, Holmes P, Cohen JD (2006) The physics of optimal decision making: A formal analysis of models of performance in two-alternative forced-choice tasks. Psychological Review 113: 700–765. 17014301 10.1037/0033-295X.113.4.700

[129] Goldman-Rakic P (1995) Cellular basis of working memory. Neuron 14: 477–485. 7695894 10.1016/0896-6273(95)90304-6

[130] Wang XJ (2001) Synaptic reverberation underlying mnemonic persistent activity. Trends in Neurosciences 24: 455–463. 11476885 10.1016/s0166-2236(00)01868-3

[131] Fransen E, Lansner A (1995) Low spiking rates in a population of mutually exciting pyramidal cells. Network: Computation in Neural Systems 6: 271–288.

[132] Lisman JE, Fellous JM, Wang XJ (1998) A role for nmda-receptor channels in working memory. Nature Neuroscience 1: 273–275. 10195158 10.1038/1086

[133] Wang XJ (1999) Synaptic basis of cortical persistent activity: the importance of nmda receptors to working memory. The Journal of Neuroscience 19: 9587–9603. 10531461 10.1523/JNEUROSCI.19-21-09587.1999 PMC6782911

[134] Durstewitz D, Seamans JK, Sejnowski TJ (2000) Dopamine-mediated stabilization of delay-period activity in a network model of prefrontal cortex. Journal of Neurophysiology 83: 1733–1750. 10712493 10.1152/jn.2000.83.3.1733

[135] Durstewitz D, Seamans JK (2006) Beyond bistability: biophysics and temporal dynamics of working memory. Neuroscience 139: 119–133. 16326020 10.1016/j.neuroscience.2005.06.094

[136] Durstewitz D, Seamans JK, Sejnowski TJ (2000) Neurocomputational models of working memory. Nature Neuroscience 3: 1184–1191. 11127836 10.1038/81460

[137] Drew MR, Simpson EH, Kellendonk C, Herzberg WG, Lipatova O, et al (2007) Transient over-expression of striatal d2 receptors impairs operant motivation and interval timing. The Journal of Neuroscience 27: 7731–7739. 17634367 10.1523/JNEUROSCI.1736-07.2007 PMC6672869

[138] Grace AA (1991) Phasic versus tonic dopamine release and the modulation of dopamine system responsivity: a hypothesis for the etiology of schizophrenia. Neuroscience 41: 1–24. 1676137 10.1016/0306-4522(91)90196-u

[139] Dreyer JK, Herrik KF, Berg RW, Hounsgaard JD (2010) Influence of phasic and tonic dopamine release on receptor activation. The Journal of Neuroscience 30: 14273–14283. 20962248 10.1523/JNEUROSCI.1894-10.2010 PMC6634758

[140] Matell MS, Berridge KC, Aldridge JW (2006) Dopamine d1 activation shortens the duration of phases in stereotyped grooming sequences. Behavioural Processes 71: 241–249. 16246504 10.1016/j.beproc.2005.09.008

[141] Body S, Cheung THC, Bezzina G, Asgari K, Fone KCF, et al (2006) Effects of d-amphetamine and doi (2,5-dimethoxy-4-iodoamphetamine) on timing behavior: interaction between d1 and 5-ht2a receptors. Psychopharmacology 189: 331–343. 17051415 10.1007/s00213-006-0575-0

[142] Cheung THC, Bezzina G, Asgari K, Body S, Fone KCF, et al (2006) Evidence for a role of d1 dopamine receptors in d-amphetamine's effect on timing behaviour in the free-operant psychophysical procedure. Psychopharmacology 185: 378–388. 16538470 10.1007/s00213-006-0339-x

[143] Cheung THC, Bezzina G, Hampson CL, Body S, Fone KCF, et al (2007) Evidence for the sensitivity of operant timing behaviour to stimulation of d1 dopamine receptors. Psychopharmacology 195: 213–222. 17668188 10.1007/s00213-007-0892-y

[144] Frederick DL, Allen JD (1996) Effects of selective dopamine d1- and d2-agonists and antagonists on timing performance in rats. Pharmacology, Biochemistry and Behavior 53: 759–764. 10.1016/0091-3057(95)02103-5 8801575

[145] Drew MR, Fairhurst S, Malapani C, Horvitz JC, Balsam PD (2003) Effects of dopamine antagonists on the timing of two intervals. Pharmacology, Biochemistry and Behavior 75: 9–15. 10.1016/s0091-3057(03)00036-4 12759108

[146] Rammsayer TH (2008) Neuropharmacological approaches to human timing. In: Grondin S, editor, The psychology of time, Bingley, UK: Emerald Group Publishing. pp. 295–320.

[147] Lotharius J, Brundin P (2002) Pathogenesis of parkinson's disease: dopamine, vesicles and alphasynuclein. Nature Reviews Neuroscience 3: 932–942. 12461550 10.1038/nrn983

[148] Smith JG, Harper DN, Gittings D, Abernethy D (2008) The effect of parkinsons disease on time estimation as a function of stimulus duration range and modality. Brain and Cognition 64: 130–143. 10.1016/j.bandc.2007.01.005 17343966

[149] Merchant H, Luciana M, Hooper C, Majestic S, Tuite P (2008) Interval timing and parkinsons disease: heterogeneity in temporal performance. Experimental Brain Research 184: 233–248. 17828600 10.1007/s00221-007-1097-7

[150] Koch G, Costa A, Brusa L, Peppe A, Gatto I, et al (2008) Impaired reproduction of second but not millisecond time intervals in parkinsons disease. Neuropsychologia 46: 1305–1313. 18215403 10.1016/j.neuropsychologia.2007.12.005

[151] Schultz W (2000) Multiple reward systems in the brain. Nature Reviews Neuroscience 1: 199–207. 11257908 10.1038/35044563

[152] Hanes DP, Schall JD (1996) Neural control of voluntary movement initiation. Science 274: 427–430. 8832893 10.1126/science.274.5286.427

[153] Ivanoff J, Branning P, Marois R (2008) fmri evidence for a dual process account of the speed-accuracy tradeoff in decision-making. Public Library of Science One 3: 1–14. 10.1371/journal.pone.0002635 PMC2440815 18612380

[154] van Veen V, Krug MK, Carter CS (2008) The neural and computational basis of controlled speed accuracy tradeoff during task performance. Journal of Cognitive Neuroscience 20: 1952–1965. 18416686 10.1162/jocn.2008.20146

[155] Miller EK (2000) The prefrontal cortex and cognitive control. Nature Reviews Neuroscience 24: 59–65. 10.1038/35036228 11252769

[156] Lo CC, Wang XJ (2006) Corticobasal ganglia circuit mechanism for a decision threshold in reaction time tasks. Nature Neuroscience 9: 956–963. 16767089 10.1038/nn1722

[157] Ditterich J (2006) Evidence for time-variant decision making. European Journal of Neuroscience 24: 3628–3641. 17229111 10.1111/j.1460-9568.2006.05221.x

[158] Eckhoff P, Wong-Lin KF, Holmes P (2009) Optimality and robustness of a biophysical decision-making model under norepinephrine modulation. The Journal of Neuroscience 29: 4301–4311. 19339624 10.1523/JNEUROSCI.5024-08.2009 PMC2750074

[159] Huk AC, Shadlen MN (2005) Neural activity in macaque parietal cortex reflects temporal integration of visual motion signals during perceptual decision making. The Journal of Neuroscience 25: 10420–10436. 16280581 10.1523/JNEUROSCI.4684-04.2005 PMC6725829

